# Exosomal miR-125b-5p derived from adipose-derived mesenchymal stem cells enhance diabetic hindlimb ischemia repair via targeting alkaline ceramidase 2

**DOI:** 10.1186/s12951-023-01954-8

**Published:** 2023-06-12

**Authors:** Jiahe Guo, Xiaofan Yang, Jing Chen, Cheng Wang, Yue Sun, Chengqi Yan, Sen Ren, Hewei Xiong, Kaituo Xiang, Maojie Zhang, Chengcheng Li, Guoyong Jiang, Xuejiao Xiang, Gui Wan, Tao Jiang, Yu Kang, Xiang Xu, Zhenbing Chen, Wenqing Li

**Affiliations:** 1 Department of Hand Surgery, Union Hospital, Tongji Medical College, Huazhong University of Science and Technology, Wuhan, 430022 China; 2 The State Key Laboratory Breeding Base of Basic Science of Stomatology (Hubei-MOST) and Key Laboratory of Oral Biomedicine Ministry of Education, School and Hospital of Stomatology, Wuhan University, Wuhan, 430022 China; 3 Department of Neurosurgery, Zhongnan Hospital of Wuhan University, Wuhan, 430071 China; 4 Department of Emergency Surgery, Union Hospital, Tongji Medical College, Huazhong University of Science and Technology, Wuhan, 430022 China; 5 Department of Hand and Foot Surgery, Huazhong University of Science and Technology Union Shenzhen Hospital, Shenzhen, 518052 China

**Keywords:** Adipose-derived mesenchymal stem cells, Exosomes, miR-125b-5p, Hindlimb ischemia, Alkaline ceramidase 2, Bioinformatics analysis

## Abstract

**Introduction:**

Ischemic diseases caused by diabetes continue to pose a major health challenge and effective treatments are in high demand. Mesenchymal stem cells (MSCs) derived exosomes have aroused broad attention as a cell-free treatment for ischemic diseases. However, the efficacy of exosomes from adipose-derived mesenchymal stem cells (ADSC-Exos) in treating diabetic lower limb ischemic injury remains unclear.

**Methods:**

Exosomes were isolated from ADSCs culture supernatants by differential ultracentrifugation and their effect on C2C12 cells and HUVECs was assessed by EdU, Transwell, and in vitro tube formation assays separately. The recovery of limb function after ADSC-Exos treatment was evaluated by Laser-Doppler perfusion imaging, limb function score, and histological analysis. Subsequently, miRNA sequencing and rescue experiments were performed to figure out the responsible miRNA for the protective role of ADSC-Exos on diabetic hindlimb ischemic injury. Finally, the direct target of miRNA in C2C12 cells was confirmed by bioinformatic analysis and dual-luciferase report gene assay.

**Results:**

ADSC-Exos have the potential to promote proliferation and migration of C2C12 cells and to promote HUVECs angiogenesis. In vivo experiments have shown that ADSC-Exos can protect ischemic skeletal muscle, promote the repair of muscle injury, and accelerate vascular regeneration. Combined with bioinformatics analysis, miR-125b-5p may be a key molecule in this process. Transfer of miR-125b-5p into C2C12 cells was able to promote cell proliferation and migration by suppressing ACER2 overexpression.

**Conclusion:**

The findings revealed that miR-125b-5p derived from ADSC-Exos may play a critical role in ischemic muscle reparation by targeting ACER2. In conclusion, our study may provide new insights into the potential of ADSC-Exos as a treatment option for diabetic lower limb ischemia.

**Supplementary Information:**

The online version contains supplementary material available at 10.1186/s12951-023-01954-8.

## Highlights

1. ADSC-Exos have the potential to facilitate muscle repair in the context of diabetes.
2. miR-125b-5p derived from ADSC-Exos played a crucial role in muscle injury.
3. The primary biological function of miR-125b-5p is achieved by targeting ACER2.
4. The AMPK signaling pathway may play a significant role in diabetic hindlimb ischemia.

**Supplementary Information:**

The online version contains supplementary material available at 10.1186/s12951-023-01954-8.

## Introduction

Lower limb ischemia is a prevalent complication of diabetes, characterized by insidious onset and longer disease duration. Clinical studies have demonstrated that collateral remodeling and angiogenesis are impaired under diabetic conditions, leading to an increased risk of lower limb ischemia [1–3]. This condition can progress to severe complications such as diabetic foot ulcers, avascular necrosis, amputation, and even death, with diabetes accounting for half of the lower limb amputations worldwide and significantly increasing mortality rates [4–7]. Conventional treatments for lower limb ischemia, such as drug therapy and surgery, have limited long-term prognoses, highlighting the urgent need for novel therapeutic strategies [8]. As the increase of diabetes continues to rise, the number of patients with lower limb ischemia is also increasing [9, 10]. Therefore, innovative therapeutic approaches are crucial to effectively address this growing health issue.

Adipose-derived mesenchymal stem cells (ADSCs) offer numerous advantages, including abundant tissue sources and multipotent differentiation capacities, which make them a promising treatment option for lower limb ischemia [8]. However, their clinical use has been limited by poor survivability in the ischemic vessel environment, difficult control of directed differentiation in vivo, and risk of microvascular embolism [11]. Recent evidence has suggested that the therapeutic effects of ADSCs may be mediated in an autocrine or paracrine manner [12]. In particular, exosomes (Exos) have attracted attention due to their ability to deliver contents such as proteins, lipids, and miRNA and play a role in intercellular communication [13]. As a cell-free strategy, exosomes from adipose-derived mesenchymal stem cells (ADSC-Exos) offer highly stable, non-immunogenic, and non-tumorigenic properties. In recent years, ADSC-Exos have been widely applied for tissue repair, including promoting diabetic wound healing, facilitating peripheral nerve regeneration, and facilitating angiogenesis in ischemic tissues [14–17]. Therefore, ADSC-Exos hold great promise as a new treatment perspective for diabetic lower limb ischemia.

MicroRNAs (miRNAs) are a class of evolutionarily conserved, single-stranded non-coding RNAs that play a vital role in regulating the transcriptome [18]. By binding to the 3′ untranslated region (UTR) of target mRNAs, miRNAs could induce regulation of gene expression [19]. Increasing evidence suggests that the enriched miRNAs in MSC-Exos are responsible for the reparative role in tissue injury, including ischemic tissue repair [20, 21]. For instance, MSC-derived exosomes carrying miR-182-5p participate in alleviating myocardial ischemia–reperfusion injury, while miR-675-abundant MSC-Exos can prevent aging-induced vascular dysfunction in mouse hindlimbs [22, 23]. Exosomal miR-126 also enhances angiogenesis by modulating the expression of genes involved in angiogenic pathways [21]. Taken together, these findings highlight the essential role of miRNAs in the therapeutic effect of MSC-Exos.

In this study, we aimed to investigate the therapeutic effect of ADSC-Exos on lower limb ischemic injury in diabetes and the underlying mechanisms. The findings demonstrate that ADSC-Exos exert its protective effects, at least in part, via the miR-125b-5p/ACER2 signaling pathway in the context of diabetic HLI. These results provide important insights into the potential clinical application of ADSC-Exos for the treatment of diabetic lower limb ischemia.

## Results

### Identification of ADSCs and ADSC-Exos

**Fig. 1:**
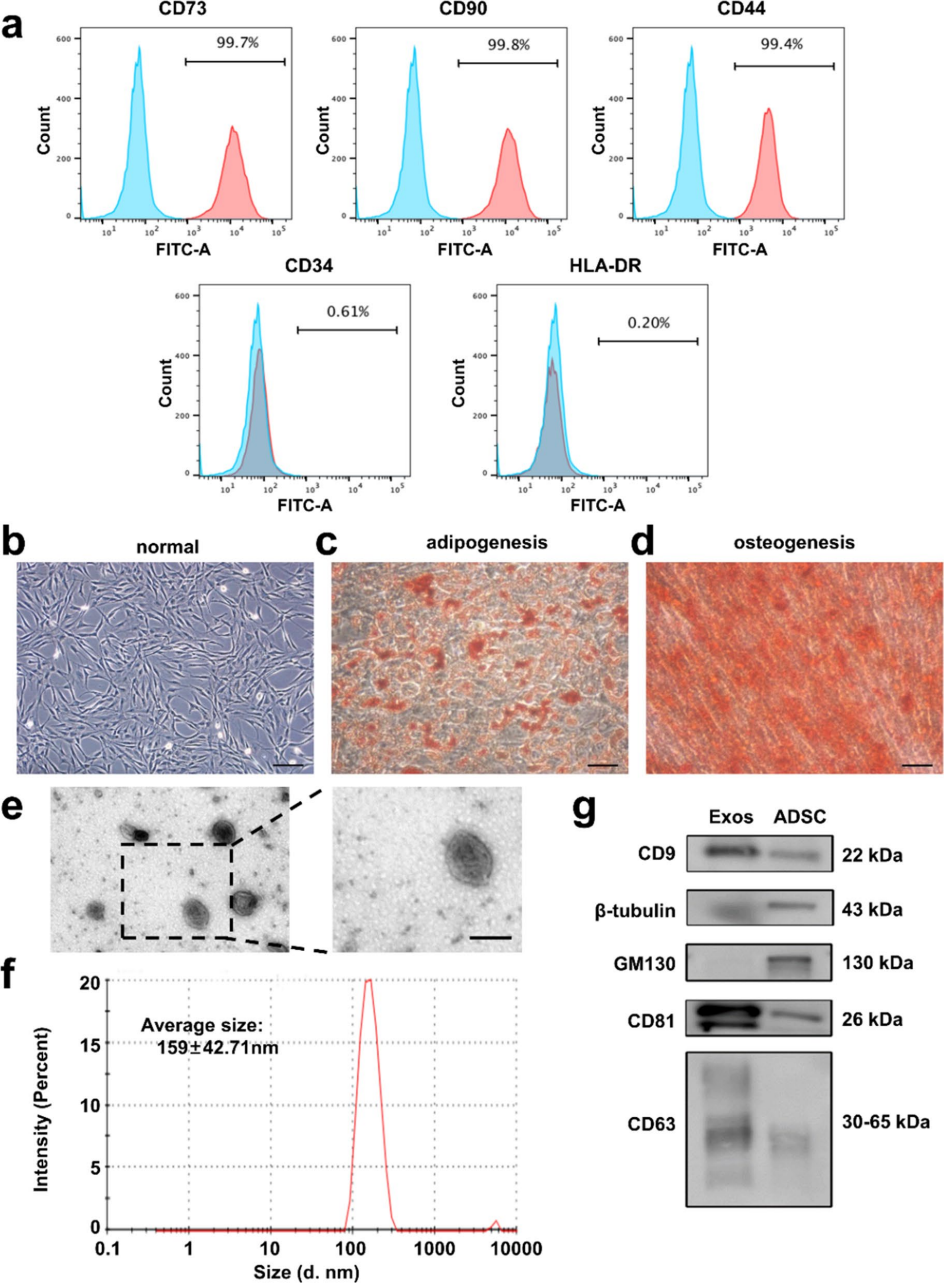
Identification of ADSCs and ADSC-Exos. **a** Flow cytometry analysis showed that ADSC were highly positive for CD73, CD90 and CD44, but negative for CD34 and HLA-DR. **b** The morphology of ADSC (Scar bar, 100 μm). **c**, **d** The typical phenotypes of osteocytes (stained with Alizarin Red) and adipocytes (stained with Oil Red O) (Scar bar, 200 μm). **e** Exosomes isolated from ADSC culture media were observed by electron microscope (Scale bar: 100 nm). **f** Measurement of ADSC-Exos population using nanoparticle tracking analysis (NTA) demonstrated a single-peaked pattern. **g** Surface markers of ADSC-Exos were confirmed by western blotting (CD9, CD63, and CD81)

Primary ADSCs were extracted from human adipose tissue and identified by the surface markers and multi-lineage differentiation potential. Flow cytometry showed almost complete positivity for CD73 (99.7%), CD90 (99.8%), and CD44 (99.4%) and no expression of CD34 and HLA-DR (Fig. 1a). ADSCs were observed typical fibroblast-like morphology (Fig. 1b). Under the mesenchymal lineage differentiation conditions, ADSCs exhibited adipogenic and osteogenic lineage phenotypes (Fig. 1c, d).

Exosomes were isolated from ADSCs culture supernatants by differential ultracentrifugation as previous protocol [15]. Transmission electron microscopy (TEM) was used to confirm the morphology of ADSC-Exos (Fig. 1e). Nanoparticle tracking analysis (NTA) showed that the mean diameter of ADSC-Exos was 159 ± 42.71 nm (Fig. 1f). Western blotting analysis revealed the exosomal markers CD9, CD63, and CD81 were expressed in ADSC-Exos, while GM130 and β-tubulin were negatively expressed (Fig. 1g).

### Internalization and protective effect of ADSC-Exos

**Fig. 2:**
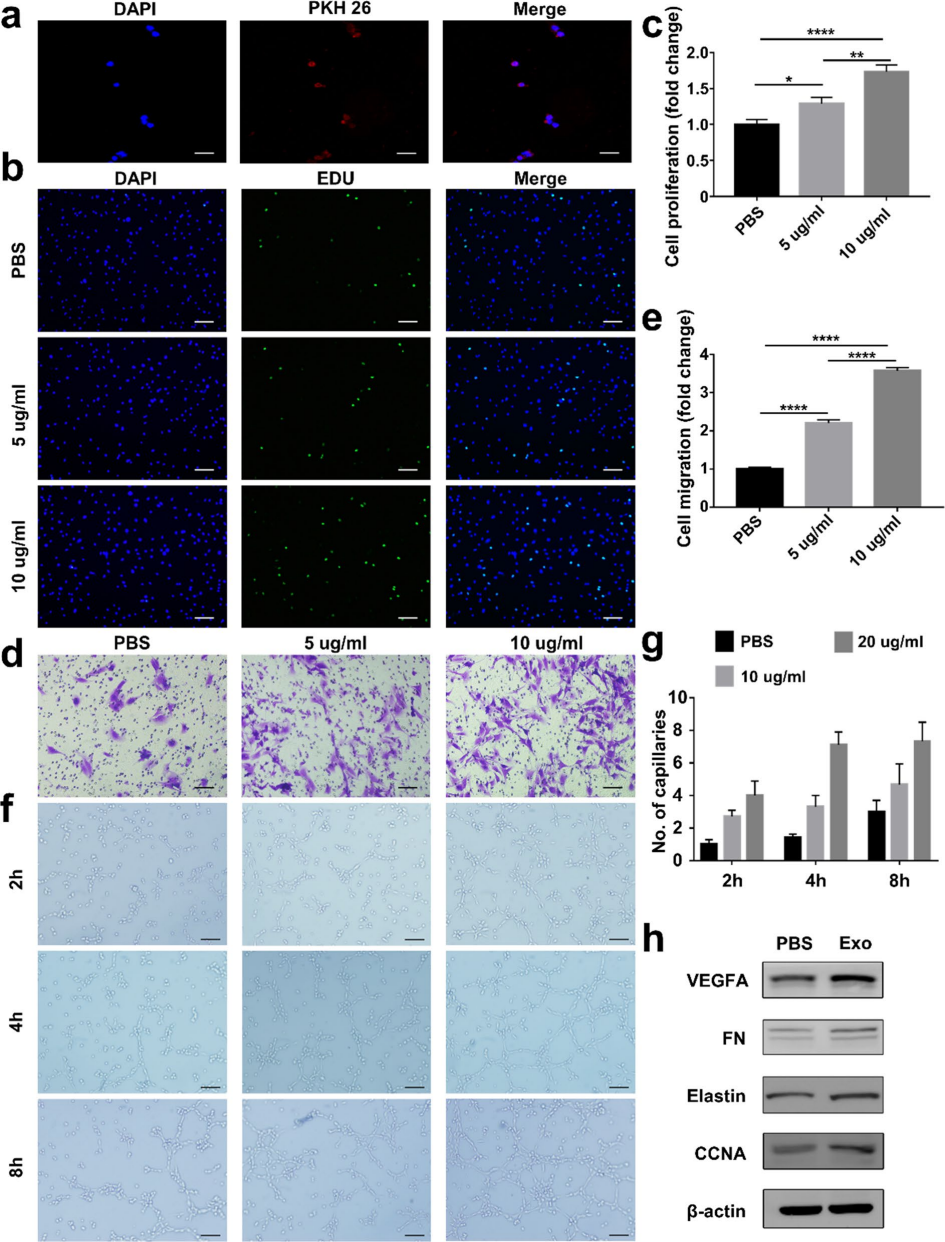
The promotion effect of ADSC-Exos. **a** Confocal images of C2C12 cells incubated with 20 μg/mL PKH26-labeled ADSC-Exos (red) for 24 h (Scale bar: 50 μm). **b** Representative micrographs show that C2C12 cells were stained with Hoechst (blue) and EdU (green) (Scale bar: 50 μm). **c** Qualification analysis of the proliferation rate. The data were collected as the six randomly chosen fields from three independent experiments (n = 5). **d** Images of migrated C2C12 cells (Scale bar: 50 μm). **e** Quantitative analysis of the migration rate of C2C12 cells. The data are expressed as six randomly chosen fields from three independent experiments (n = 5). **f** HUVECs tube formation in a Matrigel assay (Scale bar, 100 μm). **g** Qualification of the closed tubular structure shown in **f** (n = 3). **h** Protein levels in C2C12 cells treated with 20 μg/mL ADSC-Exos. *p < 0.05, **p < 0.01, ****p < 0.0001

To explore the fate of ADSC-Exos in C2C12 cells, ADSC-Exos were labeled with PKH-26 and co-cultured with C2C12 cells. Red fluorescence was observed in the cytoplasm of C2C12 cells proving that ADSC-Exos were internalized by C2C12 cells (Fig. 2a, Additional file 1: Fig. S1).

The proliferative and invasive capacities of C2C12 cells were assessed with 5-ethynyl-2´-deoxyuridine (EdU) and Transwell assay. More EdU-positive cells were observed when the C2C12 cells were treated with ADSC-Exos (Fig. 2b, c). Transwell assays exhibited that ADSC-Exos facilitated the migration potential of C2C12 cells (Fig. 2d, e). Tube formation assays were executed to evaluate the ability of ADSC-Exos to promote HUVECs angiogenesis. As expected, ADSC-Exos dramatically promoted angiogenesis (Fig. 2f, g). Additionally, ADSC-Exos treatment upregulated protein associated with proliferation, migration, and angiogenesis. After co-cultured with 20 μg/mL ADSC-Exos for 24 h, cyclin A2, VEGFA, fibronectin, and elastin were upregulated (Fig. 2h, Additional file 1: Fig. S2), but there were no differences for cyclin D1. In conclusion, these data suggest that ADSC-Exos enhance the proliferation and migration of C2C12 cells and angiogenesis of HUVECs in vitro.

### ADSC-Exos accelerated diabetic hindlimb ischemia repair

**Fig. 3:**
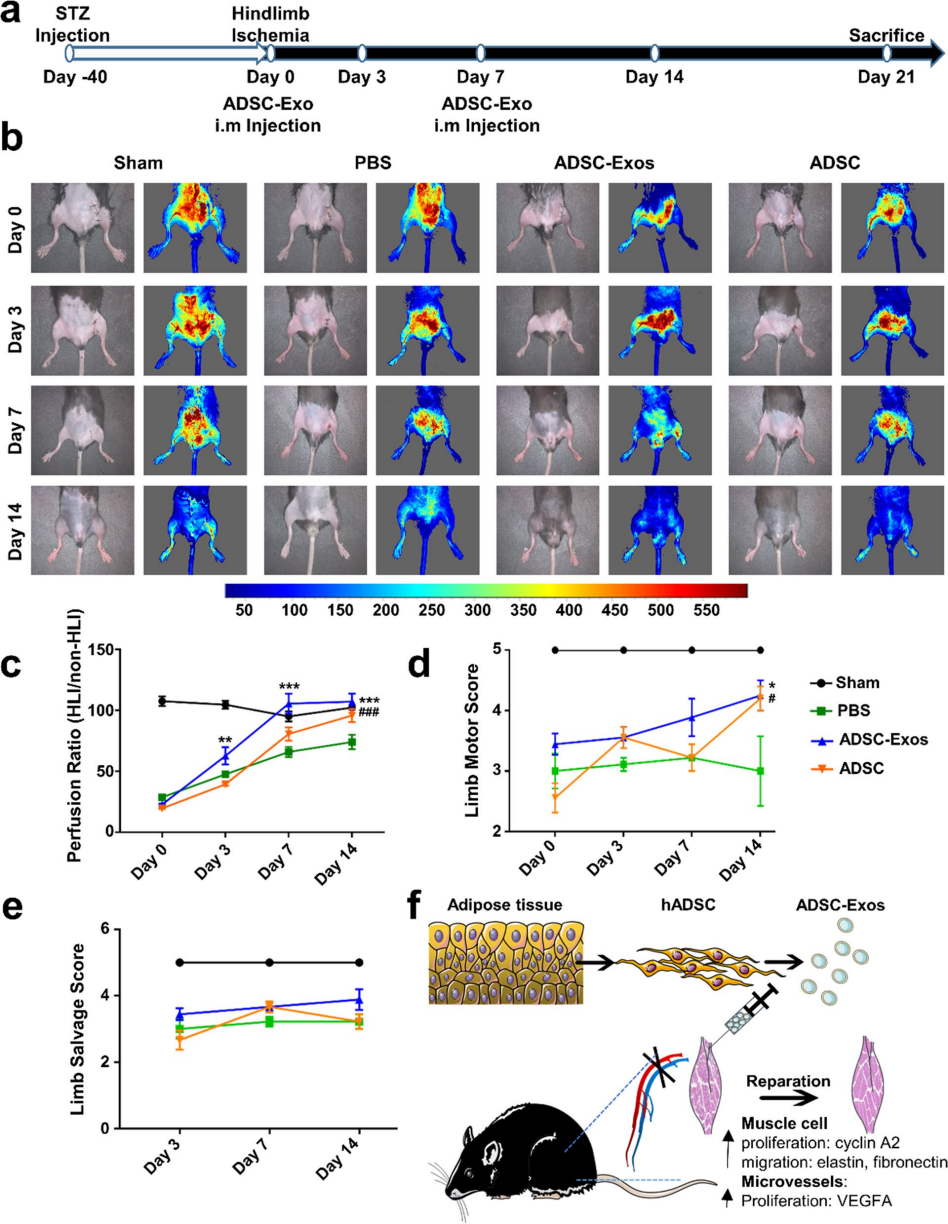
ADSC-Exos accelerated diabetic hindlimb ischemia (HLI) repair. **a** Flow chart for diabetic HLI model construction. **b** Representative image of blood perfusion in ischemic limbs. **c** Quantification of blood reperfusion ratio in four groups (n = 5, **p < 0.01, ***p < 0.001, ###p < 0.001 versus PBS). **d** Quantification of limb motor score (n = 5, *p < 0.05, #p < 0.05 versus PBS). **e** Quantification of limb salvage score (n = 5).** f** Schematic of ADSC-Exos therapy on diabetic HLI

To determine whether ADSC-Exos could promote diabetic lower limb ischemia repair, we establish a diabetic hindlimb ischemia (HLI) model (Fig. 3a). During the treatment, the perfusion ratio was higher in mice treated with ADSC-Exos and ADSCs than in untreated mice on day 7 after surgery (Fig. 3b). Moreover, the perfusion of the ADSC-Exos group was higher than that of the ADSCs group on day 3 and day 7 (Fig. 3c). After being treated with ADSC-Exos for 7 days, there was a rapid return of flow in the ischemic limbs to essentially normal levels. This evidence revealed that ADSC-Exos could accelerate HLI repair and shorten recovery duration. Additionally, there was no difference in the limb motor score among four groups, and limb loss did not occur (Fig. 3d, e). The only difference was the weak grip and the involuntary crouching in the PBS group. In conclude, these data suggested that ADSC-Exos could promote diabetic HLI repair (Fig. 3f).

**Fig. 4:**
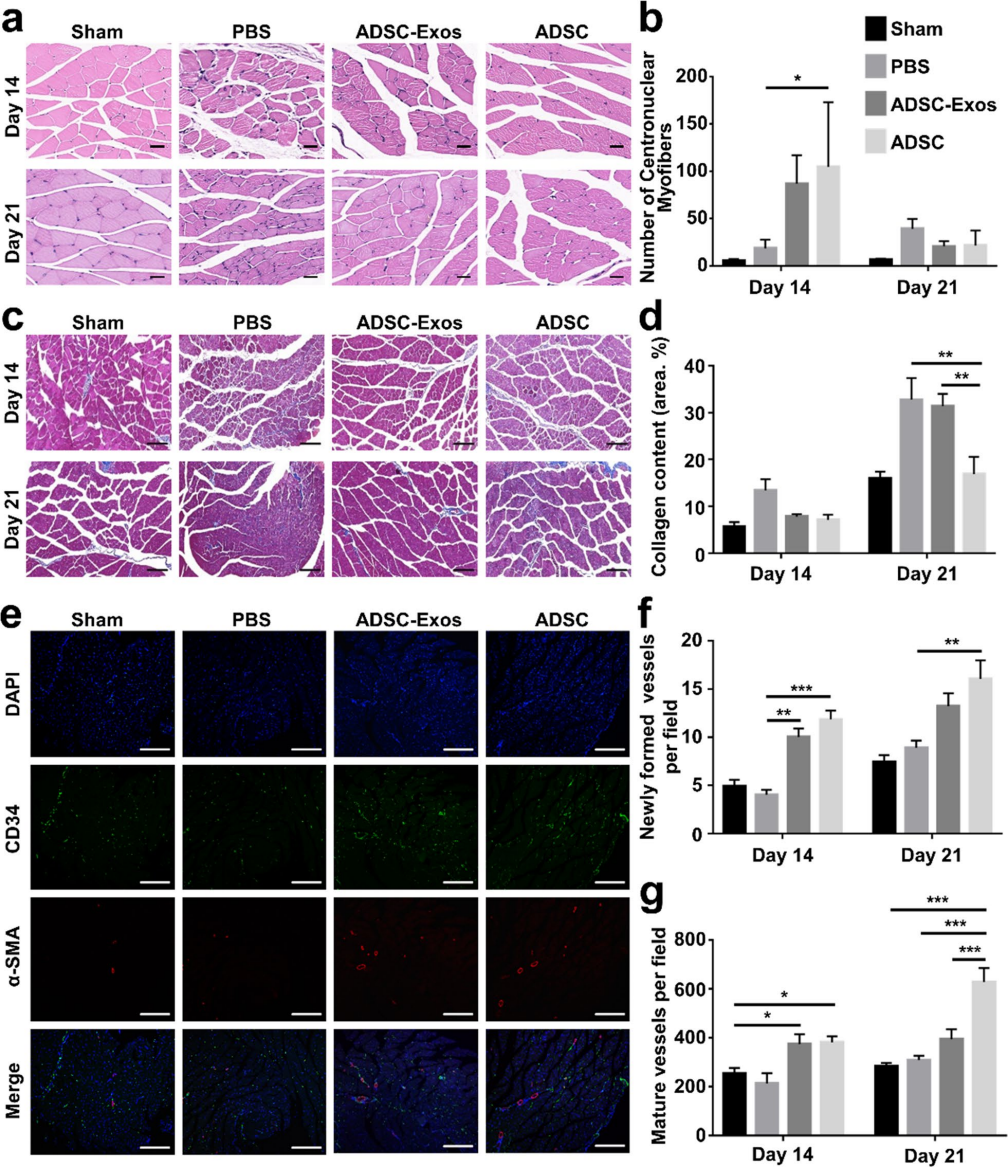
ADSC-Exos prevented the inflammatory response and enhanced neovascularization in vivo. **a** Representative HE of gastrocnemius muscles derived from the ischemic hindlimb (Scale bar: 20 µm, red arrows point to inflammatory infiltration). **b** Quantification of the number of centronuclear myofibers within the ischemic muscle at days 14 and 21 (n = 5). **c** Representative Masson staining of gastrocnemius muscles derived from ischemic hindlimb (Scale bar: 100 µm). **d** Quantification of collagen deposition at days 14 and 21 after ischemic injury (n = 5). **e** Immunofluorescent staining of gastrocnemius muscles given the different treatments at day 14 post-ischemia. Smooth muscle cells (a-SMA), endothelial cells (CD34), and cell nuclei (DAPI) were stained with red, green, and blue colors (Scale bar, 100 μm). **f** Quantification of newly formed vessels stained with green color (n = 5). **g** Quantification of mature vessels stained with red colors (n = 5, scale bar, 100 μm). *p < 0.05, **p < 0.01, ***p < 0.001

Histological analysis of muscle sections indicated that a slower progression of repair has occurred in the PBS group. Additionally, there were more proliferation cells (more centronuclear myofibers) and less collagen deposition (Masson staining) in ADSC-Exos groups compared with PBS group (Fig. 4a–d). The mean diameter of muscle fiber increased from 70.56% in the PBS group to 87.00% in the ADSC-Exos group on day 14 (Additional file 1: Fig. S3). These data suggested that ADSC-Exos significantly improve muscle fiber reparation in vivo. Immunofluorescence staining of CD34 and α-smooth muscle actin (α-SMA) were performed to observe angiogenesis in vivo (Fig. 4e, Additional file 1: Fig. S4). The number of CD34-positive capillaries and α-SMA-positive arterioles were significantly higher in the ADSC-Exos group than in the PBS group (Fig. 4f, g). Moreover, the neovascularization ability was stronger in the ADSC-Exos group than in the ADSCs group. These data suggested that ADSC-Exos could contribute to vessel formation and maturation in ischemic sites. Taken together, the above results revealed that ADSC-Exos play an essential role in the repair of diabetic HLI.

### miR-125b derived from ADSC-Exos could be delivered to C2C12 cells

**Fig. 5:**
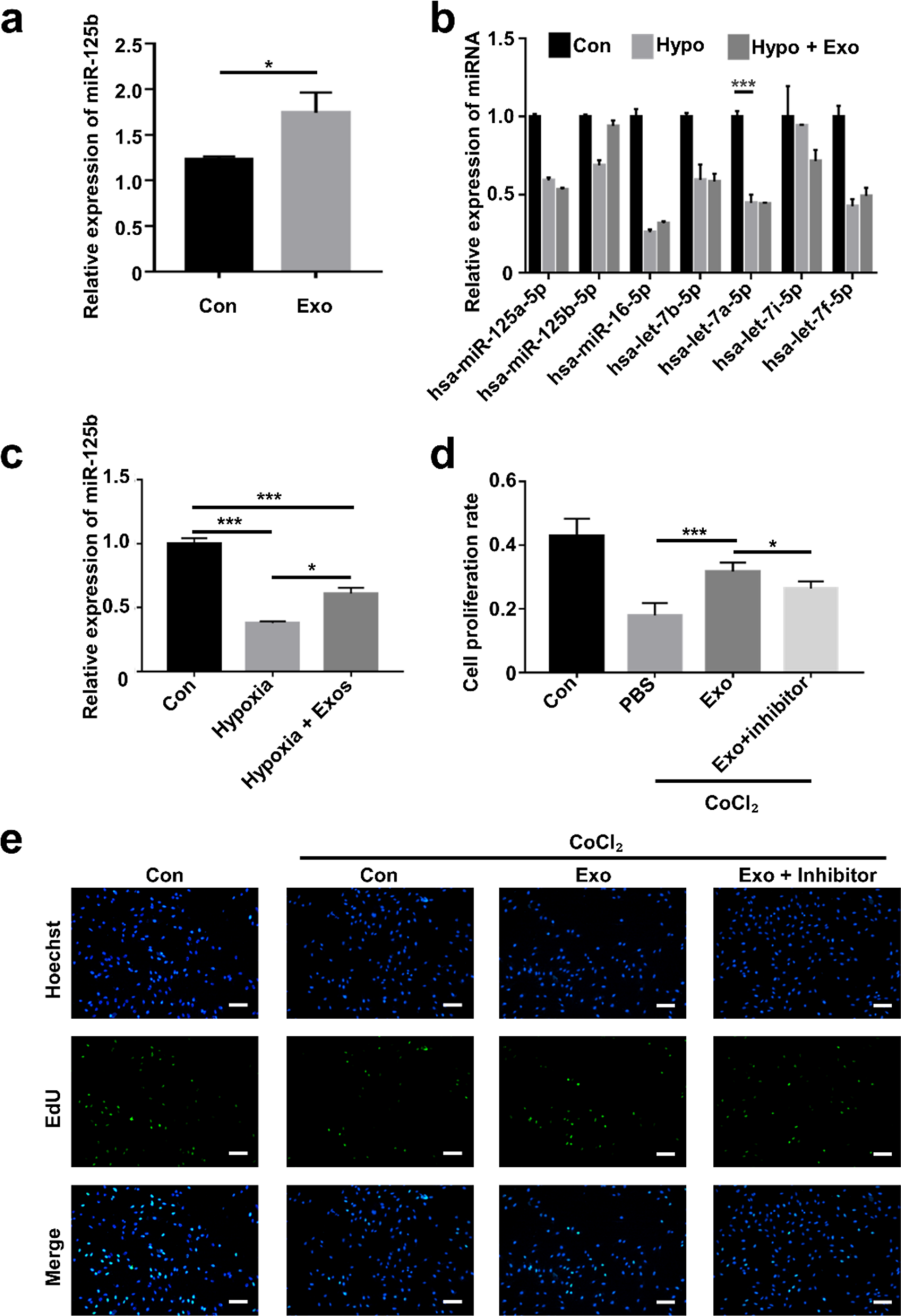
miR-125b derived from ADSC-Exos could be delivered to C2C12 cells.** a** The miR-125b-5p expression in C2C12 cells treated with ADSC-Exos. **b** The miRNA expression in C2C12 cells treated with ADSC-Exos under hypoxic conditions. **c** The miR-125b-5p expression level in C2C12 cells under hypoxic conditions. **d**, **e** EdU assay of C2C12 cells and qualification analysis of the proliferation rate (n = 5, scale bar: 50 μm). *p < 0.05, ***p < 0.001

Exosomes contain abundant miRNAs, and miRNAs have emerged as essential regulators of many biological processes. To obtain the expression profiles of miRNA and determine the mechanism responsible for the protective effects of ADSC-Exos, miRNA-seq was used in screening exosomal miRNAs. The top seven most abundant miRNAs of ADSC-Exos, including miR-125b-5p, let-7b-5p, miR-16-5p, let-7a-5p, let-7i-5p, miR-66732-5p, and miR-490-3p (Additional file 1: Fig. S5). Among them, miR-125b-5p was the most abundant miRNA in ADSC-Exos and could be delivered into C2C12 cells (Fig. 5a).

Next, we established the hypoxic cell model using cobalt chloride (CoCl_2_) for subsequent experiments and identified miR-125b-5p as the potential regulator under hypoxic conditions (Fig. 5b). ADSC-Exos could deliver miR-125b-5p into C2C12 cells under hypoxic conditions (Fig. 5c). Then, EdU assays were executed to evaluate the proliferation activity of C2C12 cells. The proliferation of cells was weakened under hypoxic condition; however, they regained the proliferative capacity after ADSC-Exos treatment (Fig. 5d, e). The protective effect of ADSC-Exos was reversed by miR-125b-5p inhibitor, suggesting that ADSC-Exos protect C2C12 cells probably through delivering miR-125b-5p.

### miR-125b regulated proliferation and migration of C2C12 cells

**Fig. 6:**
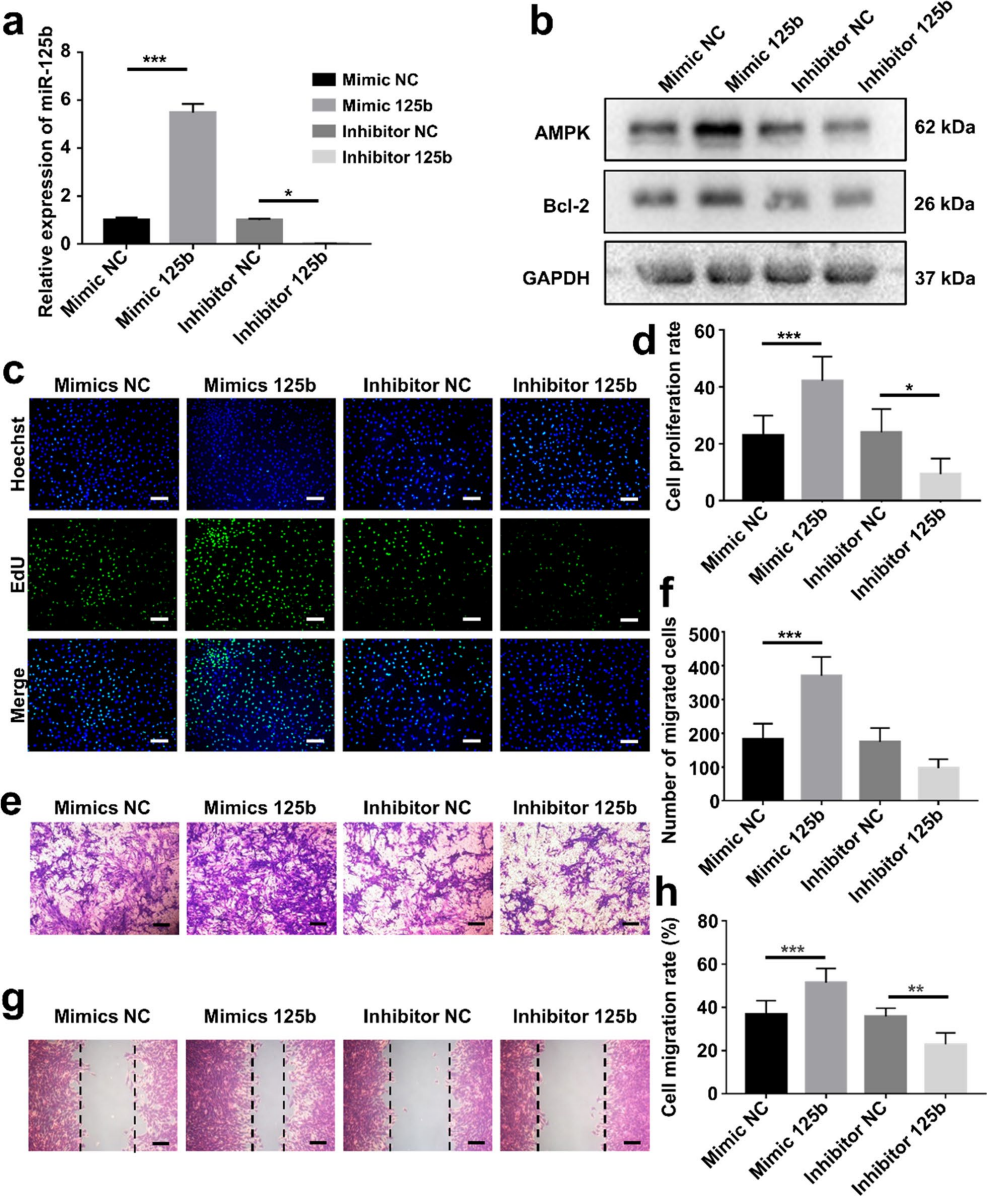
miR-125b regulated the proliferation and migration of C2C12 cells. **a** The miR-125b-5p expression in C2C12 cells treated with miR-125b-5p mimic/ inhibitor. **b** Western blotting analysis of AMPK and bcl-2 protein expression in C2C12 cells in four groups. **c** EdU assay of the proliferation rate of C2C12 cells in each group (Scale bar: 50 μm). **d** Qualification analysis of the proliferation rate (n = 5). **e** Images of migrated cells in each group (n = 5, scale bar: 50 μm). **f** Quantitative analysis of the migration rate of C2C12 cells. **g** Images depicted at 24 h after scratch (Scale bar: 50 μm). **h** Qualification analysis of the wound healing rate (n = 5). *p < 0.05, **p < 0.01, ***p < 0.001

To determine the biological effects of miR-125b, we transfected C2C12 cells with miR-125 mimic or inhibitor (Fig. 6a). In EdU assays, we observed a statistical promotion of proliferation when cells were treated with miR-125b mimic. In contrast, the miR-125b inhibitor suppressed the proliferation of C2C12 cells (Fig. 6c, d). Therefore, miR-125b was involved in the regulation of cell proliferation. Transwell assays showed that miR-125b could regulate the migration of C2C12 cells (Fig. 6e, f). Likewise, the scratch wound healing assays exhibited the same trends as Transwell assays (Fig. 6g, h). miR-125b mimic was confirmed to promote cell proliferation and migration significantly, but miR-125b acted as a suppressor in this process. Additionally, the AMPK pathway was also examined using western blot analysis. These results revealed that the miR-125b mimic enhanced AMPK expression and upregulated the expression of Bcl-2 (Fig. 6b, Additional file 1: Fig. S6). These results suggest that ADSC-Exos may be involved in regulating the AMPK signaling pathway via miR-125b.

### Bioinformation analysis of HLI in diabetic mice

We searched microarray gene expression profiling of HLI in diabetic mice and selected dataset GSE3313 for further analysis [24]. GSE3313 contains microarray data for adductor muscle in the ischemic limb from WT and Lepr db / db mice of 4 time-points (n = 3 in each timepoint). |Foldchange|> 1.5 and p < 0.05 were used as the thresholds to identify DEGs in diabetic HLI. The heatmap and volcano maps were exhibited in Additional file 1: Fig. S7a–h. Moreover, GO, KEGG and GSEA analyses were utilized to determine the functions associated with DEGs in diabetic HLI. GO analysis highlighted the top 10 biological processes (BP) terms that were mainly candidates for HLI (Additional file 1: Fig. S7i–l). A total of 332 co-DEGs in both day 7 and 14 were obtained using the R package “limma”, including 93 co-downregulated and 202 co-upregulated genes (Additional file 1: Fig. S8a). The upregulated DEGs in day 7 were mainly related to lipid metabolism and regulation of section, the downregulated DEGs were most related to skeletal system morphogenesis and immune response. Likewise, the upregulated DEGs on day 14 were mainly related to lipid metabolic process, the downregulated DEGs were most related to cell activation and inflammatory response. Lipid accumulation results in lipotoxic metabolic stress, which could promote metabolic dysfunction in skeleton muscle. Moreover, the apoptotic cells will not be removed by phagocytes timely due to the suppressed inflammation response. KEGG pathway and GO analysis were utilized to reveal the differentially expressed biological pathways in diabetic HLI (Additional file 1: Fig. S7m–p). Overlapping the KEGG and GSEA analysis produced four pathways (Additional file 1: Fig. S8b). Given that the AMPK pathway is the classical critical sensor in the regulation of energy metabolism and plays an essential role in various metabolism, we chose the AMPK signaling pathway in the subsequence research.

### Alkaline ceramidase 2 (ACER2) was the direct target of miR-125b-5p

**Fig. 7:**
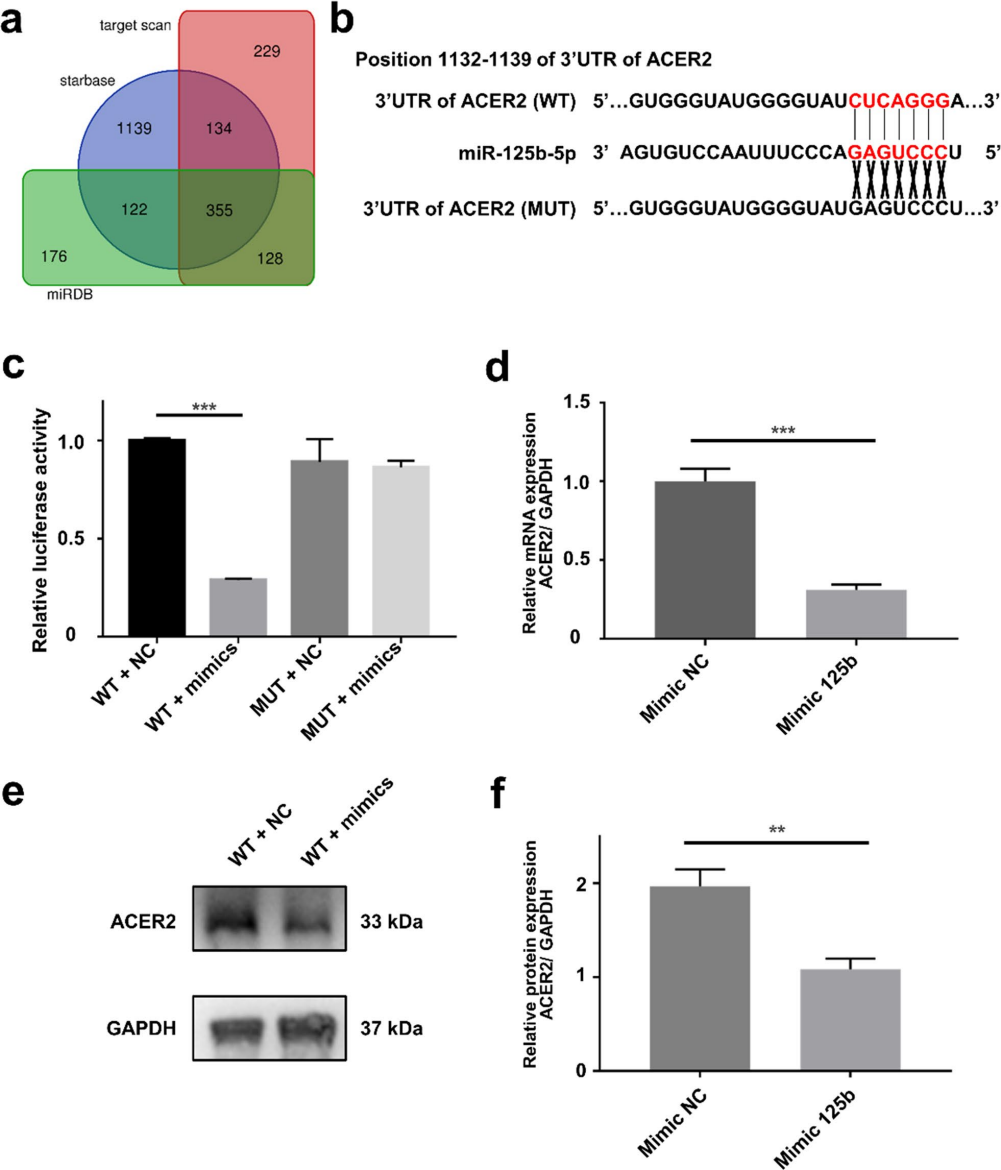
miR-125b-5p directly regulated the expression of ACER2 in C2C12 cells. **a** Venn diagram representing the number of predicted targets of miR-125b-5p. **b** Schematic of the putative binding sites or mutations of miR-125b-5p in ACER2 mRNA 3’UTR. **c** Luciferase reporter assay determined ACER2 as the target of miR-125b-5p (n = 3). **d** The mRNA expression of ACER2 in each group (n = 4). **e, f** Western blotting analysis of ACER2 protein expression in C2C12 cells. **p < 0.01, ***p < 0.001

To identify the responsible downstream mechanism of miR-125b-5p, miRDB [25], TargetScan [26], and starBase [27] were used to predict the potential target of miR-125b-5p. 355 putative miR-125b-5p targets were predicted by the three algorithms (Fig. 7a). Next, we searched for potential targets of miR-125b using the target prediction algorithm miRWalk [28] and selected ACER2 for further investigate (Additional file 1: Fig. S9). The algorithms revealed that ACER2 was a conserved target of miR-125b-5p with the pairing position of 1132–1139 in the 3’ UTR of ACER2 (Fig. 7b).

To further verify that ACER2 was the direct target of miR-125b-5p, the luciferase reporters (pmirGLO-3’UTR of ACER2-WT and ACER2-MUT) were constructed and transfected into 293 T cells. Overexpression of miR-125b-5p could suppress the luciferase activity in the ACER2-WT group (Fig. 7c). For further validation, the miR-125b-5p mimic was transfected into C2C12 cells. The ACER2 levels in miR-125b-5p transfected cells declined in both mRNA and protein levels (Fig. 7d–f). Collectively, these data proved that miR-125b-5p could suppress the expression of ACER2 by binding to the conservative complementary sequence of the ACER2 mRNA 3’UTR.

### miR-125b-5p/ACER2 axis regulated C2C12 cell function

**Fig. 8:**
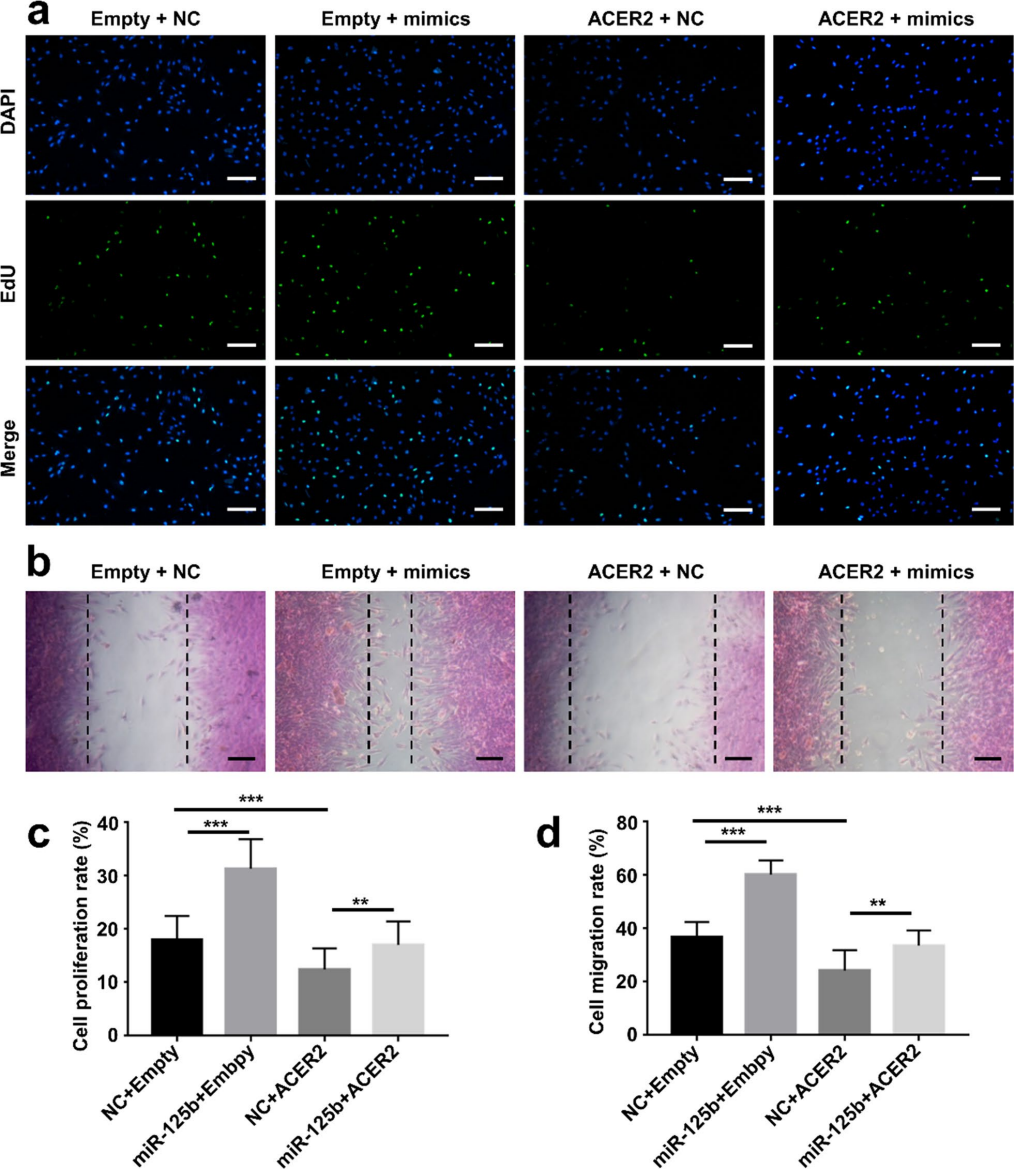
miR-125b-5p/ ACER2 axis regulated C2C12 cell function. **a** EdU assay of the proliferation rate of C2C12 cells in each group (Scale bar: 50 μm). **b** Images depicted at 24 h after scratch (Scale bar: 50 μm). **c** Qualification analysis of the proliferation rate (n = 5). **d** Qualification analysis of the wound healing rate (n = 5). **p < 0.01, ***p < 0.001

To seek out the biological function of the miR-125b-5p/ ACER2 axis, we overexpressed ACER2 in C2C12 cells. The mRNA levels of ACER2 were confirmed in C2C12 cells, suggesting that we overexpressed ACER2 in C2C12 cells through transfection of ACER2 expression plasmid (Additional file 1: Fig. S10). Then, EdU assays were executed to detect the proliferation potential of C2C12 cells. By contrast, the overexpression of ACER2 exhibited a significant anti-proliferation capacity and counteracted the protective effect of the miR-125b-5p mimic (Fig. 8a, c). Likewise, the same trend was observed in the scratch wound healing assays (Fig. 8b, d). Additionally, the expression level of AMPK and bcl-2 were suppressed by overexpression of ACER2 (Additional file 1: Fig. S11). Overall, our data demonstrated that miR-125b-5p promoted C2C12 cells’ function by targeting ACER2.

## Discussion

**Fig. 9:**
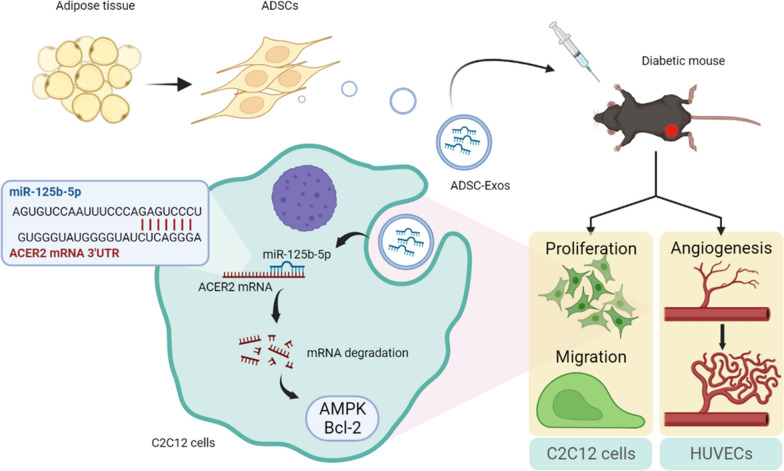
The mechanism of ADSC-Exos modulating diabetic hindlimb ischemia process (figure was created with BioRender.com)

Lower limb ischemia is a common complication of diabetes and the leading cause of non-traumatic amputation [29]. Skeletal muscle, which has high metabolic activity, is particularly vulnerable to microcirculatory dysfunction and obstruction, leading to ischemia and hypoxia injury [30–32]. Vascular endothelial damage and the accumulation of advanced glycation end products (AGEs) further exacerbate the injury under diabetic conditions [33]. In addition, hyperglycemia-induced persistent oxidant-antioxidant imbalance plays a critical role in muscle injury, leading to excessive reactive oxygen species accumulation, mitochondrial dysfunction, and apoptosis [34, 35]. In this study, we investigated the therapeutic effect of ADSC-Exos on diabetic HLI in mouse models. Our results suggest that ADSC-Exos not only promote angiogenesis but also directly contribute to muscle injury repair (Fig. 9).

Numerous studies have suggested that ADSCs can effectively treat ischemic injury in skeletal muscle. However, the application of ADSCs is limited by issues such as immune rejection and low transplantation efficacy [15]. Recently, researchers have turned their attention to exosomes as the therapeutic effects of ADSCs are mainly attributed to the paracrine factors they secrete. Exosomes play a key role in intercellular communication and immune modulation, making them a promising therapeutic option for clinical implementation [13]. Studies have shown that exosomes secreted by MSCs can stimulate the expression of angiogenesis-related genes, enhance microvessel density, and improve blood perfusion in ischemic limbs [36]. Furthermore, ADSC-Exos have been found to promote vascular regeneration and accelerate diabetic wound healing by increasing microvessel density in the wound bed [15]. In our study, we demonstrated that ADSC-Exos can promote endothelial cell angiogenesis. Moreover, we demonstrated that ADSC-Exos improve cell function of C2C12 cells and promote muscle regeneration. And in vivo assays showed that ADSC-Exos could restore revascularization and rescue ischemic limbs under diabetic conditions.

Recent researches have highlighted the importance of miRNAs in regulating stem cells and their involvement in various processes such as migration, apoptosis, and cell cycle regulation. They have also been implicated in different signaling pathways and the repair of ischemic injury. For example, miR-126-3p from stem cell exosomes was shown to promote angiogenesis and repair ischemic tissue, while exosomal miR-21 prevented cardiomyocyte apoptosis in ischemic cardiac disease [21, 37]. Other studies have demonstrated that MSC-Exos could promote tubular repair and ameliorate acute kidney injury through the modulation of the cell cycle arrest and apoptosis via miR-125b/p53 pathways [38]. Additionally, miRNA-31-5p was found to enhance endothelial cell functions, promoting angiogenesis and enhancing diabetic wound healing [39]. In our study, we focused on the exosomal miR-125b-5p in ADSCs and its effect on C2C12 cell function under ischemic conditions. Our findings suggest that miR-125b-5p may regulate apoptosis via the AMPK signaling pathway and could represent a promising therapeutic target for ischemic muscle injury treatment.

ACER2 has been identified as a key target of HIF-2α in modulating ceramide catabolism and affecting cell survival, making it a promising therapeutic target for a range of diseases [40–42]. Hypoxia has been shown to promote the expression of ACER2, which is consistent with our bioinformatics analysis data. In addition, ACER2 appears to play a regulatory role in vessel repair. Overexpression of ACER2 has been shown to rescue HIF-2α-deficiency-induced exacerbation of atherosclerosis [42]. Meanwhile, as the transcriptional target of p53, ACER2 is involved in autophagy and regulates cell survival and migration [43]. However, it's worth noting that overexpression of ACER2 has been shown to increase ROS production, and its role in regulating ROS is conserved among eukaryotes [41, 44]. In our study, we confirmed increased ACER2 levels in the diabetic HLI models and demonstrated that overexpression of ACER2 suppressed cell proliferation and migration, while this trend could be reversed by miR-125b-5p. These findings suggest that ACER2 may represent a promising target for the treatment of ischemic muscle injury, particularly in diabetic patients.

In this study, we have identified a potentially significant role for the miR-125b-5p/ACER2 pathway in promoting the repair of ischemic muscle injury. Our findings indicate that ADSC-Exos have the potential to promote endothelial and C2C12 cell function, as well as effectively repair ischemic muscle injury in vivo. Through sequencing analysis, we have discovered that miR-125b-5p, which derived from ADSC-Exos, may play a crucial protective role. By utilizing bioinformatics analysis and experimental validation, we have identified ACER2 as a target gene of miR-125b-5p. Our investigations have also confirmed the association between the AMPK signaling pathway and diabetic HLI. However, it is worth noting that we cannot exclude the possibility that other miRNAs in ADSC-Exos contribute to the observed therapeutic effects, and further research is needed to verify this.

Nevertheless, our research had some shortcomings. The AMPK signaling pathway may play an essential role in diabetic HLI, which while notable was not invested in depth. Increased ACER2 levels in diabetic HLI may lead to ROS-induced oxidative injury and related to the p53-dependent apoptosis pathways. We also lacked further investigate in the function of ACER2 in diabetic HLI. Additional studies are needed to address these issues in future research.

## Conclusion

Taken together, ADSC-Exos could accelerate the reparation of diabetic HLI. miR-125b-5p derived from ADSC-Exos can be transferred into C2C12 cells and promote cell proliferation and migration by suppressing ACER2 overexpression. These findings may provide important insights into the potential clinical application of ADSC-Exos for the treatment of diabetic lower limb ischemia.

## Method

### Isolation of ADSC and cell culture

Subcutaneous adipose tissue from healthy donor sites from patients who received flap repair were collected. The isolation and culture of ADSCs was according to the previous protocol in laboratory [45]. In brief, the fresh adipose tissue was cut into pieces and digested with collagenase type I (Sigma, USA). The cell precipitation was re-suspended and then filtered with a 70 μm filter (Corning, USA). After re-centrifugation, the cell precipitate was re-suspended in the Dulbecco’s modified Eagle’s medium (DMEM, Gibco, USA) consisting of 10% fetal bovine serum (FBS, Serapro, USA). The ADSCs were passaged 3 to 8 times for experiments.

Human umbilical vein endothelial cells (HUVECs) (#GDC166, CCTCC) and C2C12 cells (#GDC175, CCTCC) were purchased from the China Center for Type Culture Collection (CCTCC, Wuhan, China). HUVECs and C2C12 cells were cultured in culture medium supplemented with 10% fetal bovine serum, 100 μg/mL of streptomycin, and 100 U/mL of penicillin in a 5% CO_2_ humidified atmosphere.

### Isolation and characterization of ADSC-Exo

ADSCs were cultured in pretreated culture medium consisting of 10% exosome‐free FBS. The exosome‐free FBS used for culturing ADSCs was ultracentrifuged at 120,000 *g* for 12 h to get rid of extracellular vesicles. The cell supernatant was collected every 48—72 h starting at passage 3 to 8 when ADSCs reached 85–95% confluence. The supernatant of ADSC was obtained and centrifuged at 1000*g* for 10 min and 3000*g* for 25 min successively to eliminate dead cells and debris. The supernatants were moved into Amicon®Ultra-15 Centrifugal Filter (Millipore, USA), and centrifuged at 13,000*g* for 50 min. After that, supernatants were centrifuged at 120,000*g* for 90 min and 70 min separately. The pellets were resuspended in phosphate‐buffered saline (PBS) and stored at -80 ℃. Nanoparticle tracking analysis (NTA, Beckman Coulter, USA) and transmission electron microscope (Hitachi, Japan) were adapted to detect the qualification of ADSC-Exos and Pierce BCA Protein Assay Kit (Aspen, China) was adapted to detect the protein level.

### Intracellular uptake of ADSC-EXOs

ADSC-Exos were labeled with red fluorescent dye (PKH26, Sigma, USA). For internalization assay, C2C12 cells were seeded in the 35-mm confocal dishes and co-cultured with labeled ADSC-Exos. And the nucleus was stained with DAPI (Aspen, China) according to the manufacturer’s instructions. The laser scanning confocal microscope was adapted for observing the internalization of the ADSC-Exos.

### Cell function assays

#### Cell proliferation, migration, and scratch wound healing assays

For proliferation assay, 5-ethynyl-2´-deoxyuridine (EdU) (Beyotime, China) was adapted according to the manufacturer’s instructions. C2C12 cells were planted in 96-well tissue culture plates. After culturing for 24 h, the cell medium was replaced and 5 or 10 μg/mL of ADSC-Exos or PBS was added.

For migration assay, C2C12 cells were seeded in 24-well Transwell chambers (Corning, USA). Cell suspension was added to the top compartment and treated with PBS, 5 or 10 μg/mL ADSC-Exos. After incubation at 37 °C for 24 h, the chambers were removed and the cells migrating to the lower surface were stained with 0.1% crystal violet and pictured (Solarbio, China). Data were expressed as an average of cells per field that migrated through micropores.

The scratch wound healing assay was used to analyze the migration capacity of C2C12 cells. The cells were seeded in 12-well culture plates. The confluent monolayer of cells was scratched using a 200-μL pipette tip and then washed with PBS. The cells were then cultured in culture medium containing 2% FBS for 24 h. The wells were captured and analyzed using ImageJ.

#### Tube formation assay in vitro

HUVEC cells were seeded with PBS, 10 and 20 μg /mL ADSC-Exos in 48-well culture plates that pre-coated with 130 μL Matrigel Basement Membrane Matrix (BD Biosciences, USA). Tube formation was recorded microscopically at 2, 4 and 8 h incubation.

### Western blot

Equal amount of total protein (30–60 μg) was separated by SDS-PAGE (Beyotime Biotechnology, China), then transferred to the PVDF membrane (Millipore, USA). Afterward, the membranes were incubated overnight with primary antibodies. Then incubated with HRP-conjugated antibody (Aspen, China) for 1 h. Afterwards membranes were incubated with Immobilon ECL substrate kit (Millipore, USA) for 1 min and detected by BioSpectrum 600 Imaging System (UVP, USA).

### Model of diabetic HLI in vivo

All animal experiments were approved by the Animal Care Committee of Tongji Medical College. Male C57BL/6 mice (6–8 weeks) were used in the study. Type I diabetes was induced by intraperitoneal injection of 50 mg/kg streptozotocin (STZ) for 5 consecutive days, blood glucose was monitored for 2 weeks thereafter. And experiments were executed 4 weeks after confirmation of hyperglycemia status (glucose level > 16.7 mmol/L). Both the femoral artery and femoral vein in the right hindlimb were ligated at a high position, including all the small vessels to ensure ischemia [46, 47]. Mice were divided into four groups randomly: sham surgery, PBS, ADSC-Exos (100 μg) and ADSCs (5 × 10^5^ /kg). All treatments were injected into the ischemic hindlimb in 100 μL PBS at 4 different locations in adductor and gastrocnemius muscle after the surgery on Day 0 and Day 7.

### Scoring for limb functional recovery

Laser-Doppler Perfusion Imaging (LDPI) was measured at 0, 3, 7, and 14 days after posterior limb ischemia surgery. The tissue perfusion was measured by LDPI (Moor Instruments, UK), blood flow was measured in ischemic and non-ischemic limbs, and the ratio of two measurements was calculated. The hindlimb flow signal was expressed as the ratio of left (ischemic) to right (nonischemic) to avoid data variations that may be caused by ambient light and temperature. On Day 3, 7, 14 and 21 after surgery, motor function was assessed semi-quantitatively by the established scoring system (1: no limb use; 2: no foot use, limb use only; 3: restricted foot use; 4: no active toe use (spreading), foot use only; and 5: unrestricted limb use). And necrosis scores (1: limb amputation; 2: foot amputation; 3: toe(s) amputation; 4: necrosis, nail loss only; 5: full recovery) were used to grade the degrees of hindlimb impairment and ischemic damage [21].

### Histological analysis and immunofluorescence staining

Both ischemic and non-ischemic gastrocnemius muscle tissues were collected on Day 14 and Day 21 for histological analysis. The muscle tissue of the ischemic limb was fixed with 4% paraformaldehyde. Then tissues were embedded in paraffin and cut into 8-μm-thick longitudinal sections after dehydrated with a series of graded ethanol. The sections were stained with hematoxylin and eosin (H&E) for histological analysis. Masson staining was calculated to evaluate collagen accumulation in the ischemic area. The sections were incubated with α-SMA and CD34 antibody (Abcam, USA) overnight at 4 ℃. After washing with PBS three times, incubate the sections with the second antibody (Aspen, China) at room temperature for 1 h. The images were taken by microscope and analyzed with ImageJ.

### Transfection

The miR-125b-5p mimic, miR-125b-5p inhibitor, and the relevant negative controls (mimic NC and inhibitor NC) were obtained from Guangzhou RiboBio Co., Ltd. (RiboBio). C2C12 cells were transfected using riboFECT™CP Reagent according to the manufacturer’s instruction. The plasmids for overexpressing were purchased from Genomeditech Biotechnology (Shanghai, China). Cell transfection was performed with the NEOFECT™ DNA transfection reagent according to the manufacturer’s protocol. After transfected for 48 h, the cells were processed for further assays.

### RNA isolation and RT-PCR

RNA was extracted from cells and exosomes using a total RNA isolation kit (Vazyme, China) and Ultrapure RNA Kit (CW0581M, CWBIO), respectively according to the manufacture’s instruction. miRNA was reversely transcribed into cDNA using the miRNA 1st strand cDNA synthesis kit (Accurate Biotechnology (Hunan) Co., Ltd, China). The AceQ qPCR SYBR Green Master Mix was then used to carry out the qRT-PCR assay (Vazyme, China). RT-PCR was performed on Bio-Rad CFX Maestro (Bio-Rad, USA). The relative expression levels of targeted genes were calculated using the 2^−ΔΔCt^ method and normalized to β-actin and U6. The primer sequences were presented in Additional file 1: Table S1.

### Dual-luciferase report gene assay

Wuhan AUGCT Biotechnology co., LTD. created sequences that correspond to the 3’UTR of ACER2 mRNA and contain either wild-type (WT) or mutated (MUT) miR-125b binding sequences. To create the ACER2 3’-UTR reporter constructs, these sequences were cloned into XhoI and SalI restriction sites of the pmirGLO luciferase reporter vector. The C2C12 were seeded in 24-well plates and incubated for 24 h before transfection. Then the cells were co-transfected with miRNAs (mimic NC and miR-125b-5p mimic) and prirGLO luciferase reporter vector containing the WT of MUT 3’UTR of ACER2. After transfected for 48 h, the cells were collected, and luciferase was detected using Firefly &Renilla Luciferase Report Assay Kit (Meilunbio, China).

### Statistics

Quantified results are expressed as the mean ± standard errors. GraphPad Prism v7.0 software (Graphpad Software, USA) were adapted to analyze the data. Comparison between two groups was performed with unpaired Student t test. For group > 2, one-way or two-way ANOVA with Bonferroni post hoc test was used. Statistical significance was accepted at P < 0.05 (*p < 0.05, **p < 0.01, ***p < 0.001, ****p < 0.0001).

## Supplementary Information

**Additional file 1: Figure S1.** Internalization of ADSC-Exos. **Figure S2.** Relative protein expression. **Figure S3. **Mean fiber diameter in each group. **Figure S4.** Immunofluorescent staining of gastrocnemius muscles given the different treatments at day 21 post-ischemia (scale bar, 100 μm). **Figure S5.** Read counts of miRNA in ADSC-Exos. **Figure S6.** Relative protein expression of AMPK and Bcl-2. **Figure S7.** The bioinformation analysis of diabetic HLI. The heatmap (a, c, e, g) and volcano maps (b, d, f, h) on day 0, 1, 7, and 14. i, j The biological processes regulated in GO analysis on day 7. k, l The biological processes regulated in GO analysis on day 14. m, n The KEGG pathway upregulated and downregulated on day 7. o, p The KEGG pathway upregulated and downregulated on day 14. **Figure S8**. a The co-DEGs in both day 7 and 14. b The GSEA analysis of diabetic HLI. **Figure S9.** The potential targets of miR-125b. **Figure S10.** The relative mRNA expression of ACER2. **Figure S11.** a Western blotting analysis of AMPK and bcl-2 protein expression in C2C12 cells in four groups. b, c Relative protein expression of AMPK and Bcl-2. **Table S1.** Sequences used in qRT-PCR.

## Data Availability

All data sources could be available to readers on request.

## Abbreviation

ADSCs: Adipose-derived mesenchymal stem cells
MSCs: Mesenchymal stem cells
HLI: Hindlimb ischemia
Exos: Exosomes
ADSC-Exos: Exosomes from adipose-derived mesenchymal stem cells
miRNA: MicroRNA
UTR: Untranslated region
miRNA-seq: MiRNA sequencing
PBS: Phosphate‐buffered saline
TEM: Transmission electron microscopy
NTA: Nanoparticle tracking analysis
STZ: Streptozotocin
LDPI: Laser-Doppler Perfusion Imaging
HE: Hematoxylin and eosin
EdU: 5-Ethynyl-2´-deoxyuridine
WT: Wild-type
MUT: Mutated
ACER2: Alkaline ceramidase 2
ROS: Reactive oxygen species
CoCl_2_: Cobalt chloride
AMPK: Adenosine 5ʹ-monophosphate (AMP)-activated protein kinase
