# Supplementary material for: Exosomal miR-125b-5p derived from adipose-derived mesenchymal stem cells enhance diabetic hindlimb ischemia repair via targeting alkaline ceramidase 2
Source: J Nanobiotechnology. 2023 Jun 12;21:189. doi: 10.1186/s12951-023-01954-8 (PMC10259056; doi:10.1186/s12951-023-01954-8)
Supplement: Supplementary file 1 — Additional file 1: Figure S1. Internalization of ADSC-Exos. Figure S2. Relative protein expression. Figure S3. Mean fiber diameter in each group. Figure S4. Immunofluorescent staining of gastrocnemius muscles given the different treatments at day 21 post-ischemia (scale bar, 100 μm). Figure S5. Read counts of miRNA in ADSC-Exos. Figure S6. Relative protein expression of AMPK and Bcl-2. Figure S7. The bioinformation analysis of diabetic HLI. The heatmap (a, c, e, g) and volcano maps (b, d, f, h) on day 0, 1, 7, and 14. i, j The biological processes regulated in GO analysis on day 7. k, l The biological processes regulated in GO analysis on day 14. m, n The KEGG pathway upregulated and downregulated on day 7. o, p The KEGG pathway upregulated and downregulated on day 14. Figure S8. a The co-DEGs in both day 7 and 14. b The GSEA analysis of diabetic HLI. Figure S9. The potential targets of miR-125b. Figure S10. The relative mRNA expression of ACER2. Figure S11. a Western blotting analysis of AMPK and bcl-2 protein expression in C2C12 cells in four groups. b, c Relative protein expression of AMPK and Bcl-2. Table S1. Sequences used in qRT-PCR. [file 12951_2023_1954_MOESM1_ESM.docx]

**Table S1. Sequences used in qRT-PCR.**

| Symbol | Target sequence |
| --- | --- |
| miR-125a-5p | TCCCTGAGACCCTTTAACCTGT |
| miR-125b-5p | TCCCTGAGACCCTAACTTGTGA |
| let-7a-5p | GTGAGGTAGTAGGTTGTATAG |
| let-7i-5p | TGAGGTAGTAGTTTGTGCTGTT |
| miR-16-5p | TAGCAGCACGTAAATATTGGC |
| miR-4728-3p | CATGCTGACCTCCCTCCTGC |
| let-7b-5p | TGAGGTAGTAGGTTGTGTGGTT |
| let-7f-5p | GGTGAGGTAGTAGATTGTATAG |
| U6 | CCTGCTTCGGCAGCACA |
| ACER2 forward | TGTGGCATATTCTCATCTGCCT |
| ACER2 reverse | CAATAAAAGCCCATTTCTCGCTG |
| GAPDH forward | GGAGCGAGATCCCTCCAAAAT |
| GAPDH reverse | GGCTGTTGTCATACTTCTCATGG |

# Additional Figure


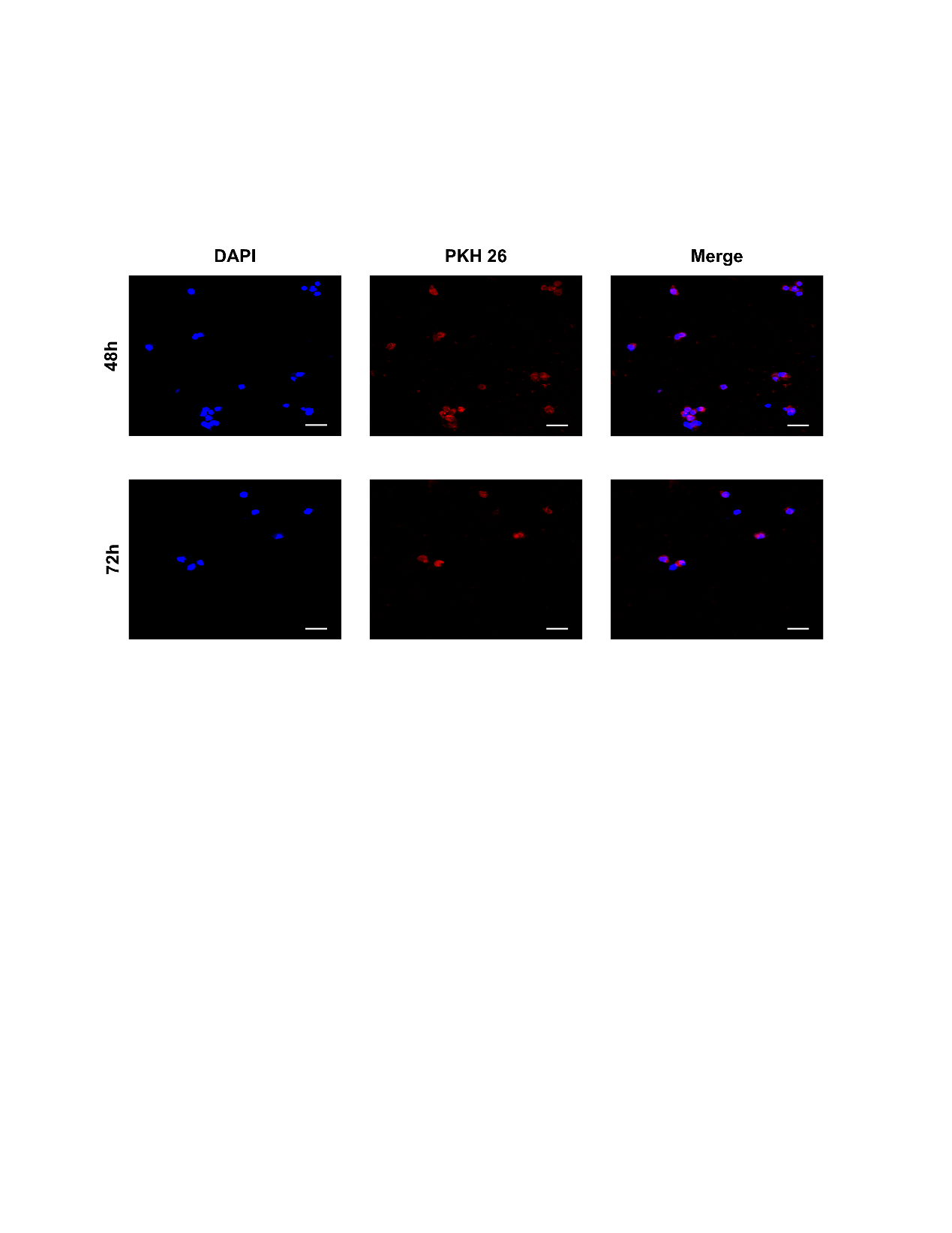


**Figure S1.** Internalization of ADSC-Exos (Scale bar: 50 μm).


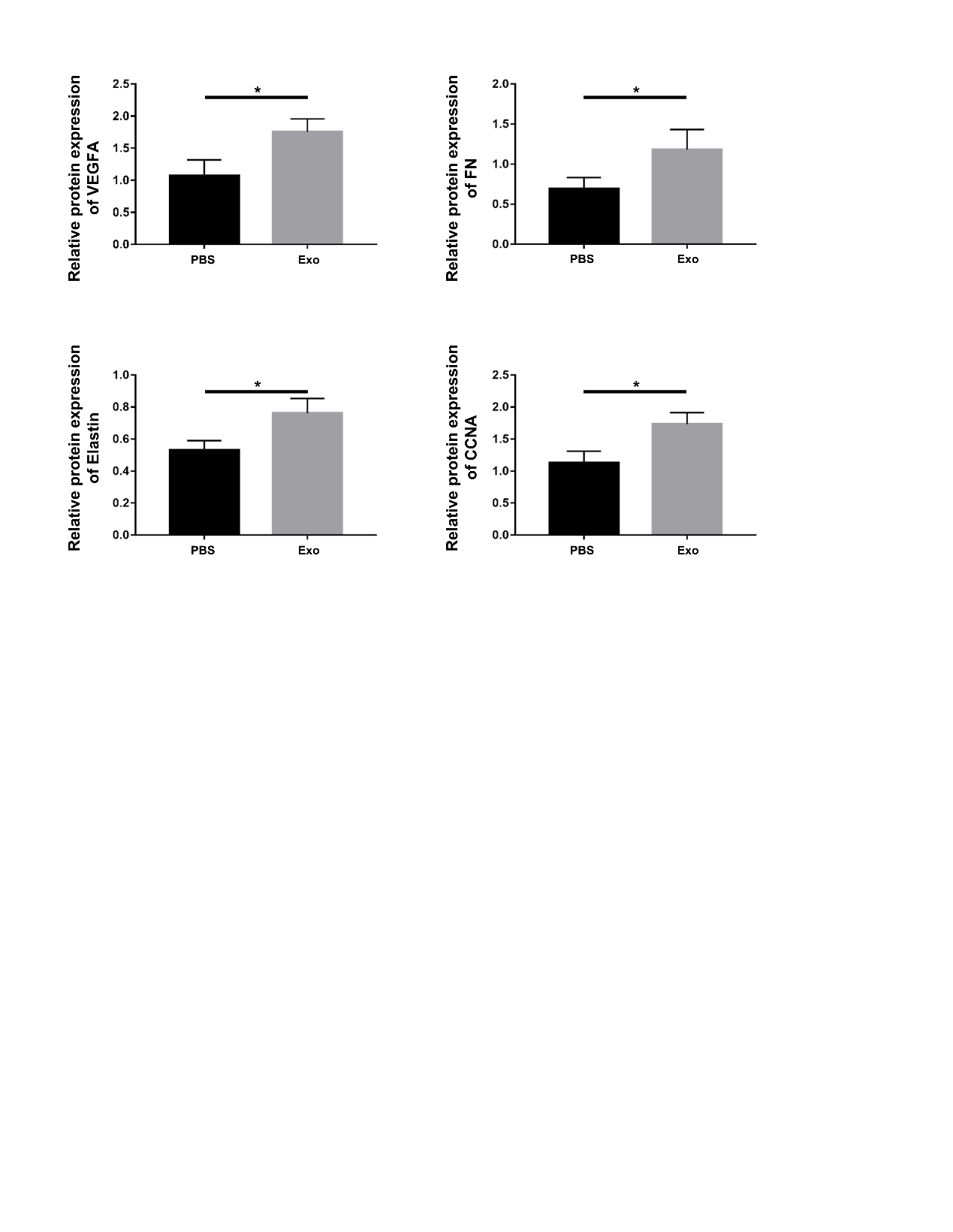


**Figure S2.** Relative protein expression.


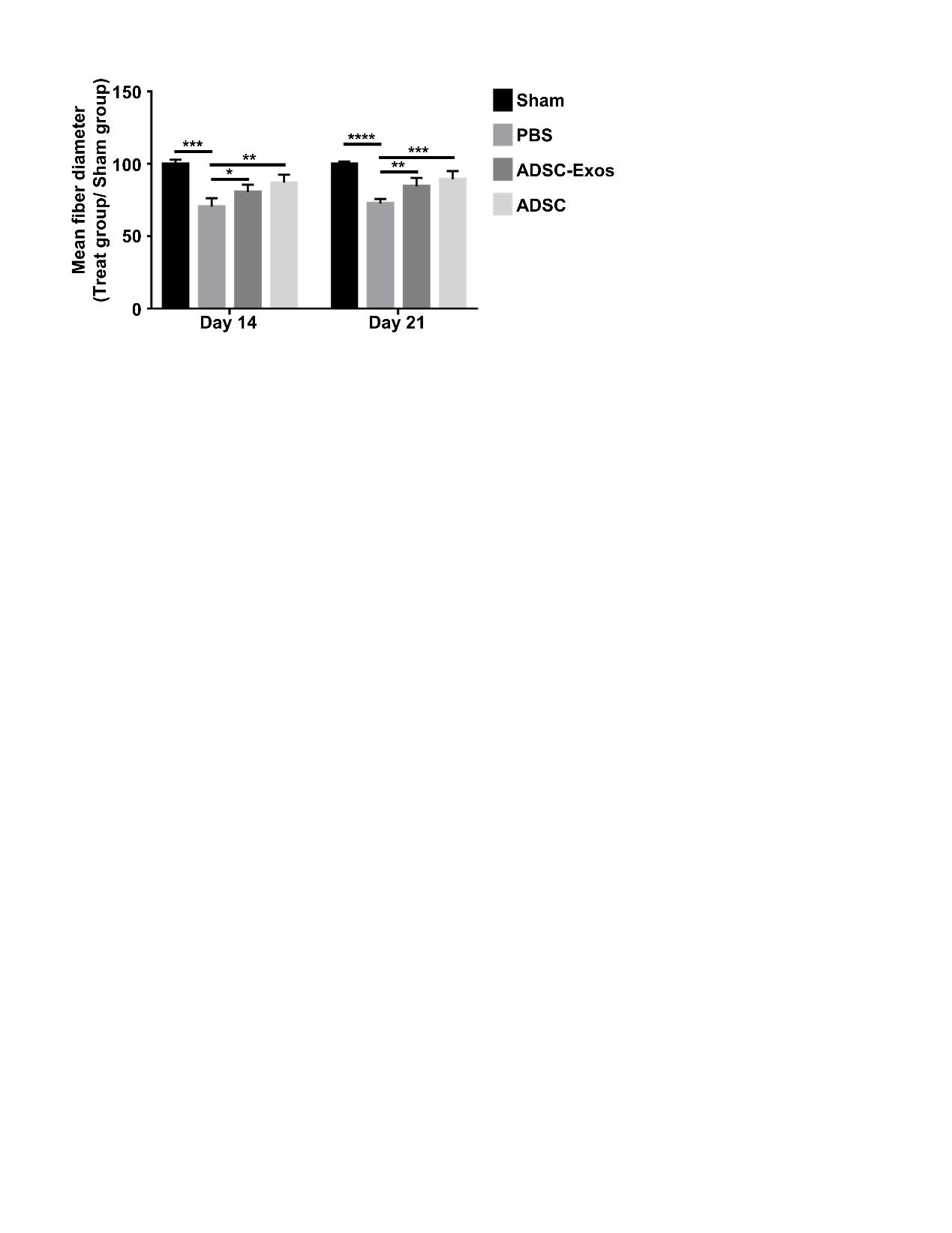


**Figure S3.** Mean fiber diameter in each group.


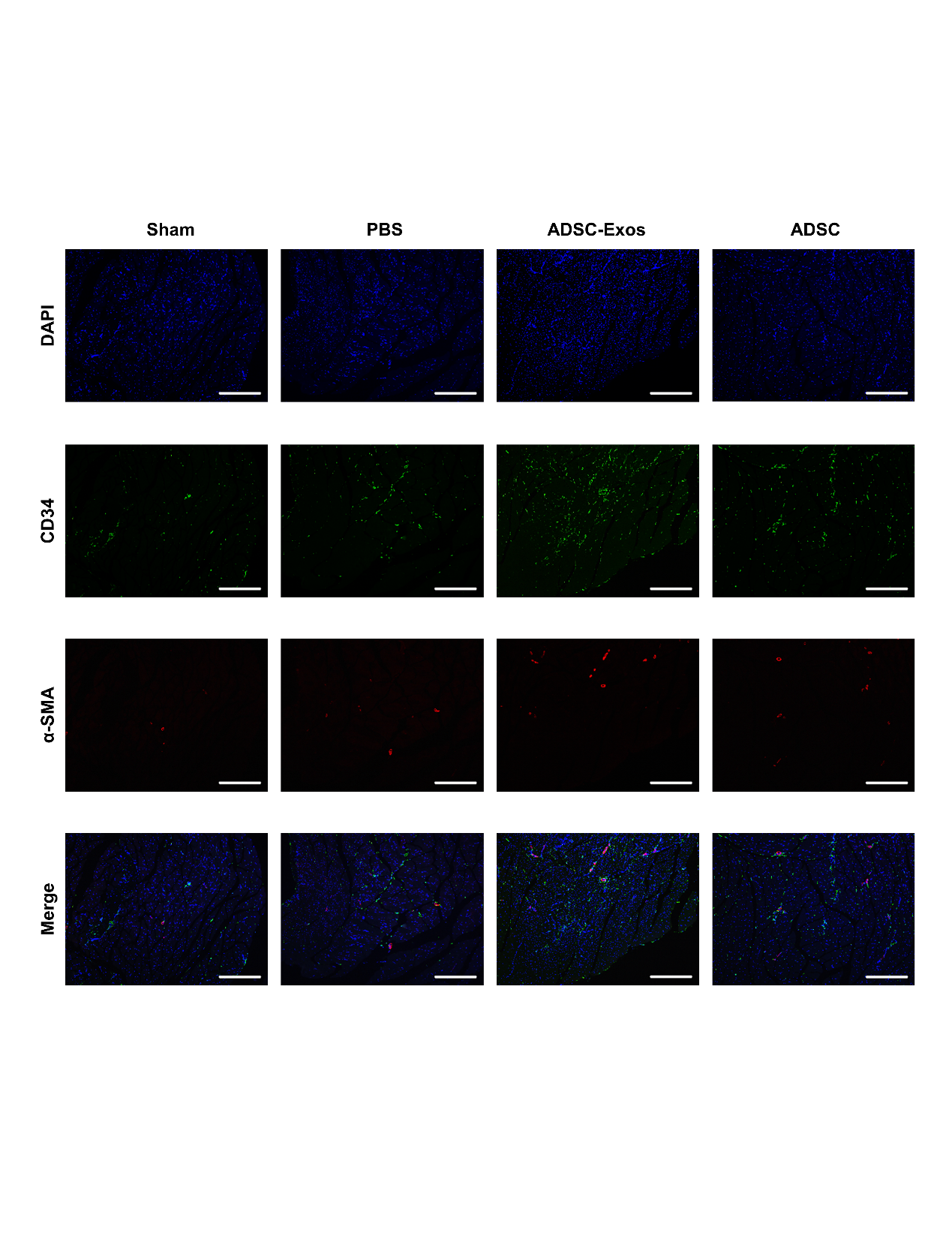


**Figure S4.** Immunofluorescent staining of gastrocnemius muscles given the different treatments at day 21 post-ischemia (scale bar, 100 μm).


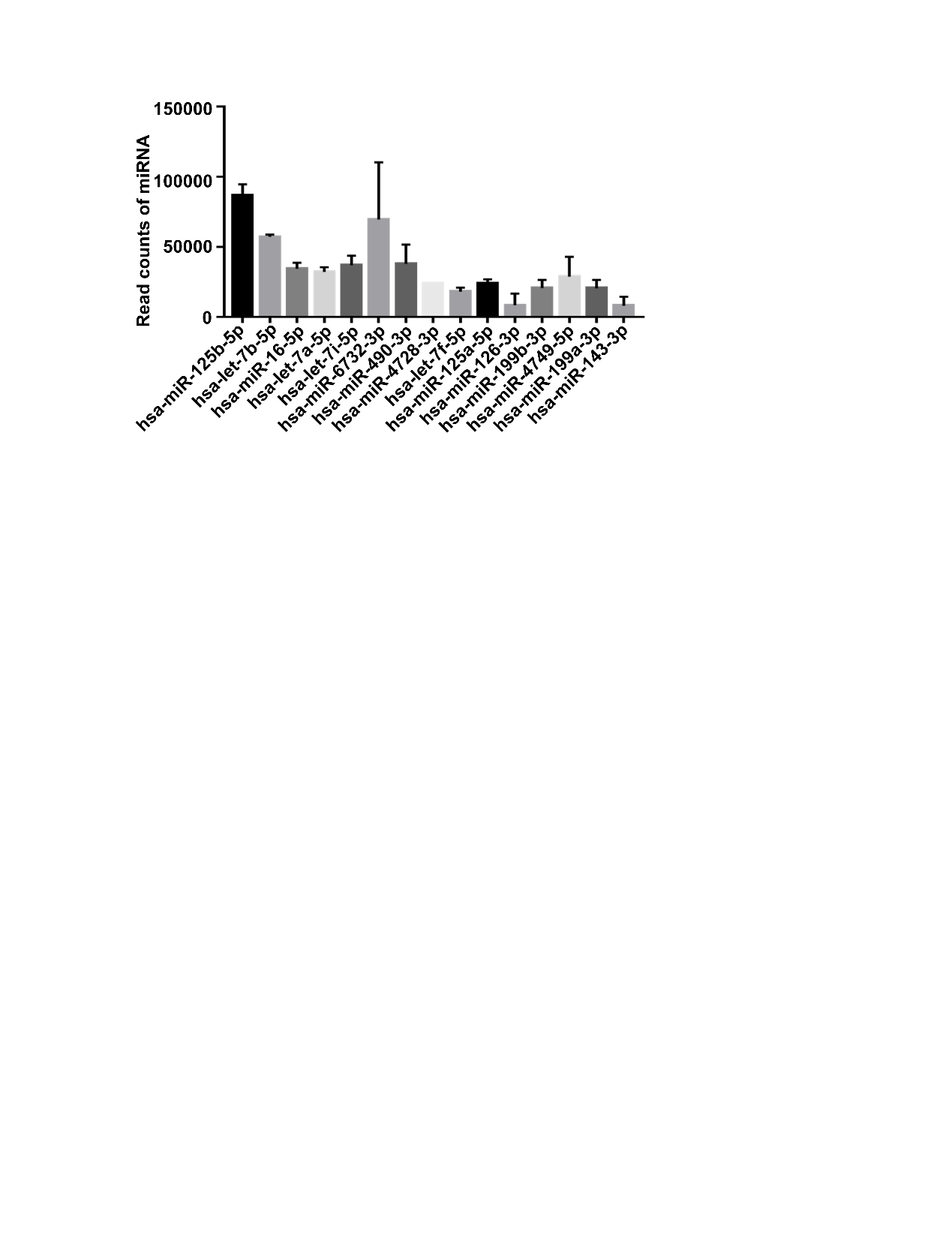


**Figure S5.** The 15 most abundant miRNAs of ADSC-Exos.


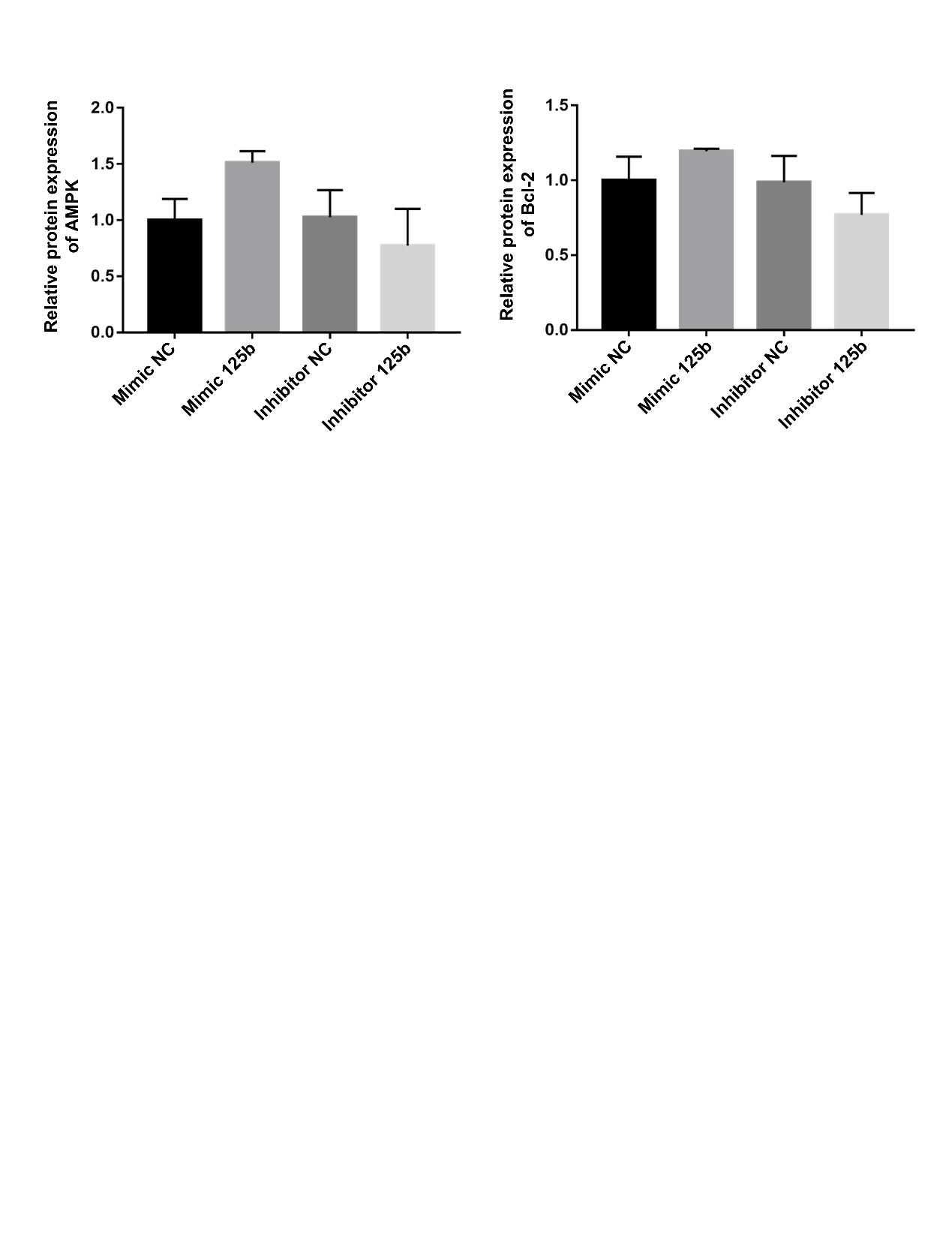


**Figure S6.** Relative protein expression of AMPK and Bcl-2.


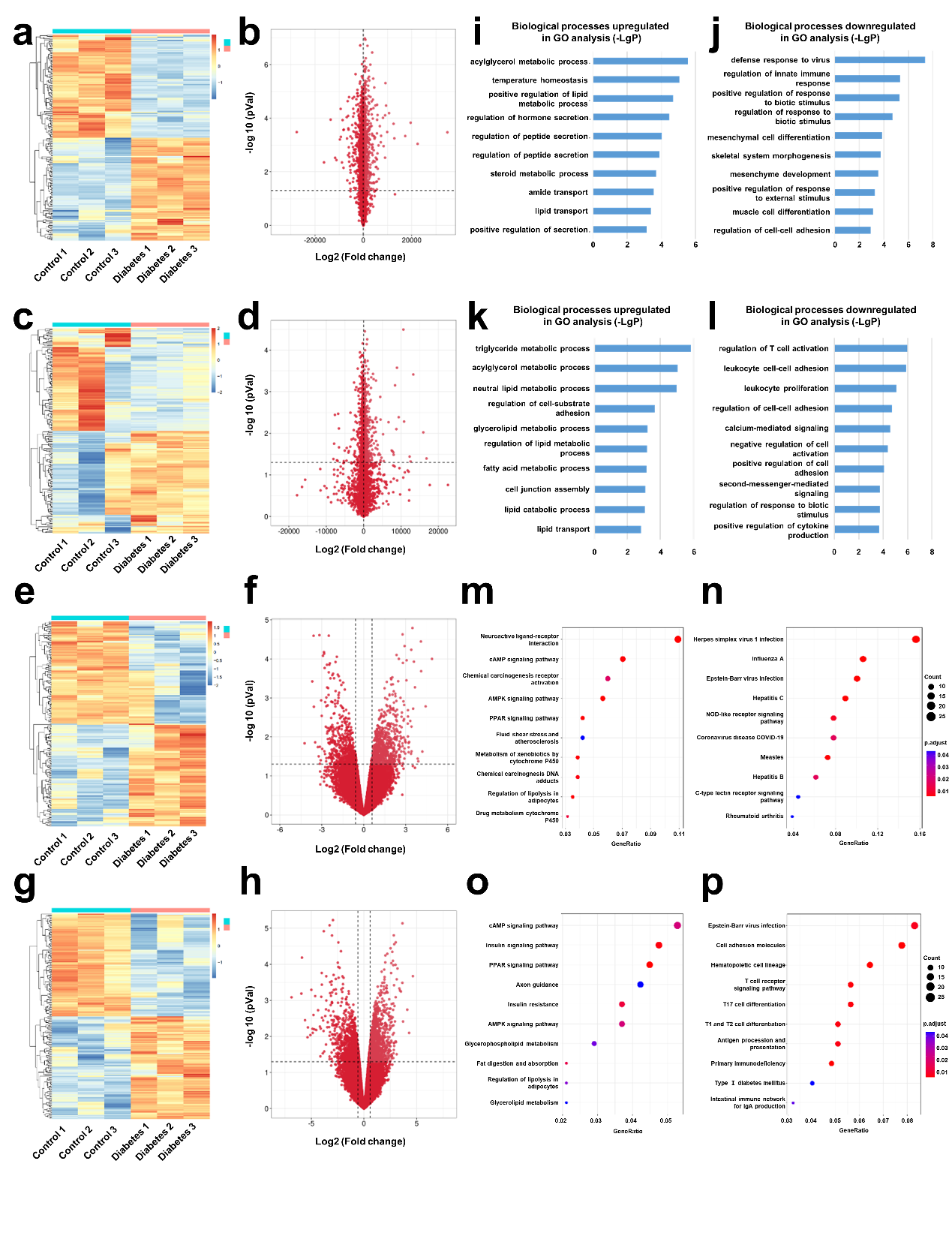


**Figure S7.** The bioinformation analysis of diabetic HLI. The heatmap (**a, c, e, g**) and volcano maps (**b, d, f, h**) on day 0, 1, 7, and 14. **i, j** The biological processes regulated in GO analysis on day 7. **k, l** The biological processes regulated in GO analysis on day 14. **m, n** The KEGG pathway upregulated and downregulated on day 7. **o, p** The KEGG pathway upregulated and downregulated on day 14.


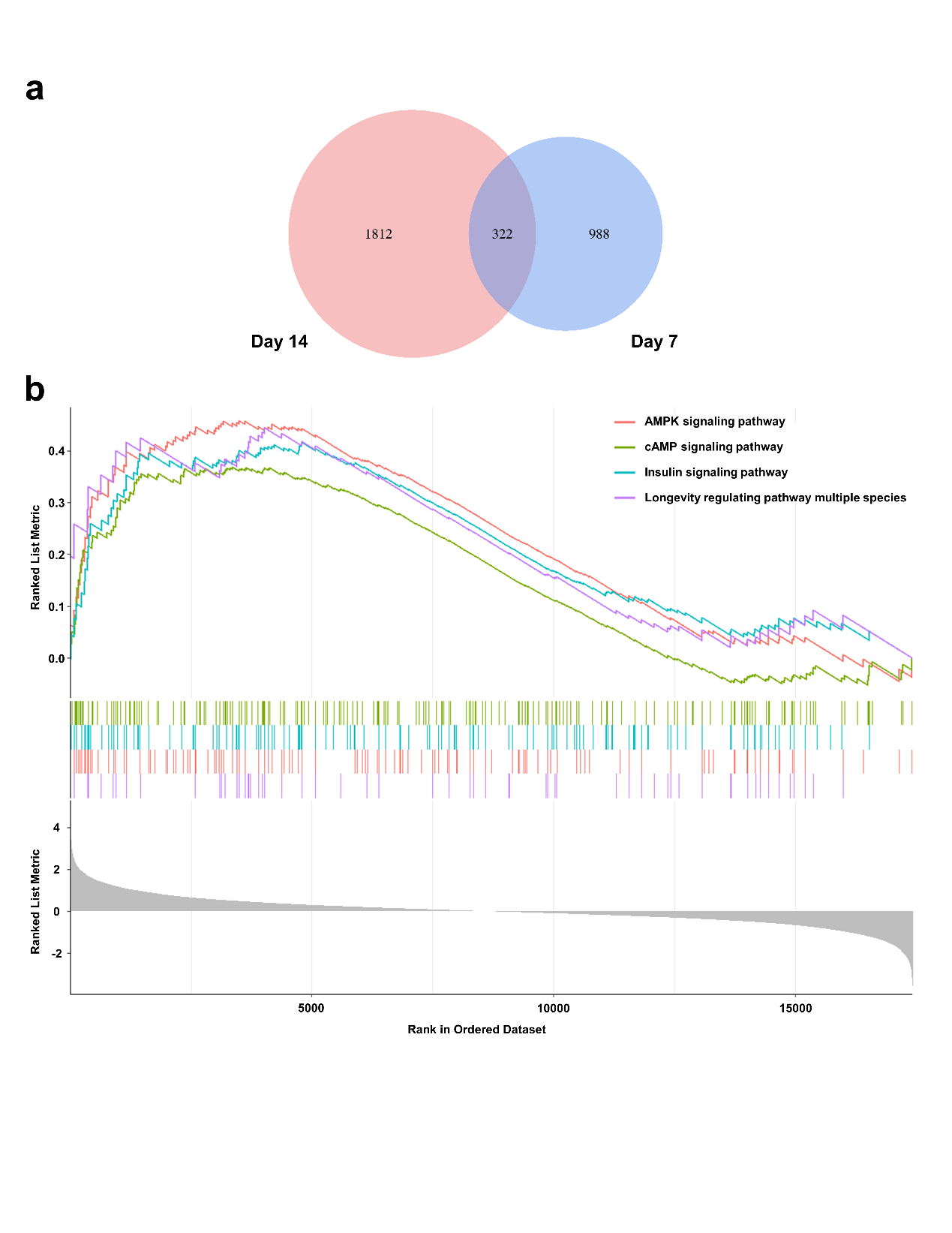


**Figure S8. a** The co-DEGs in both day 7 and 14. **b** The GSEA analysis of diabetic HLI.


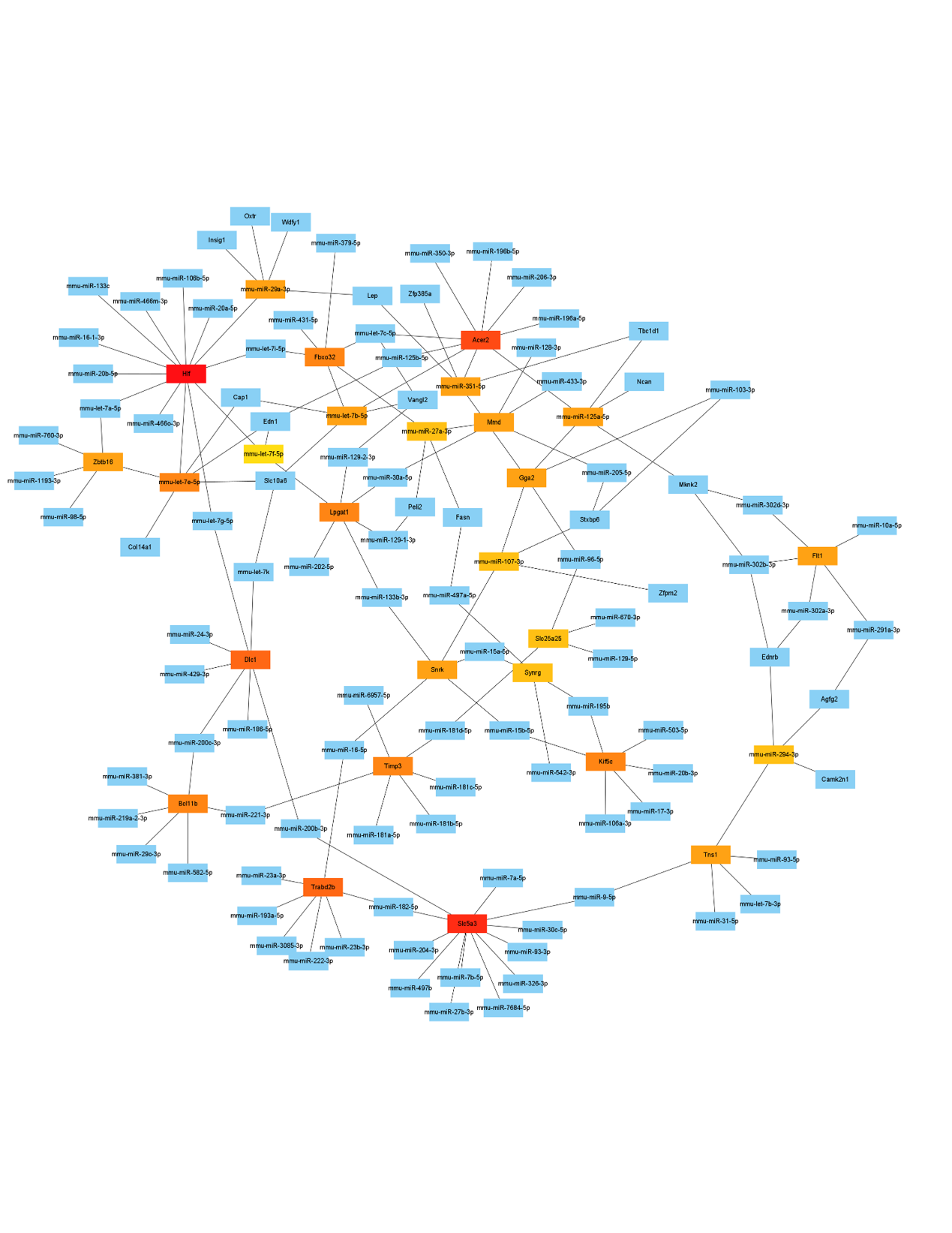


**Figure S9.** The potential targets of miR-125b using the target prediction algorithm miRWalk..


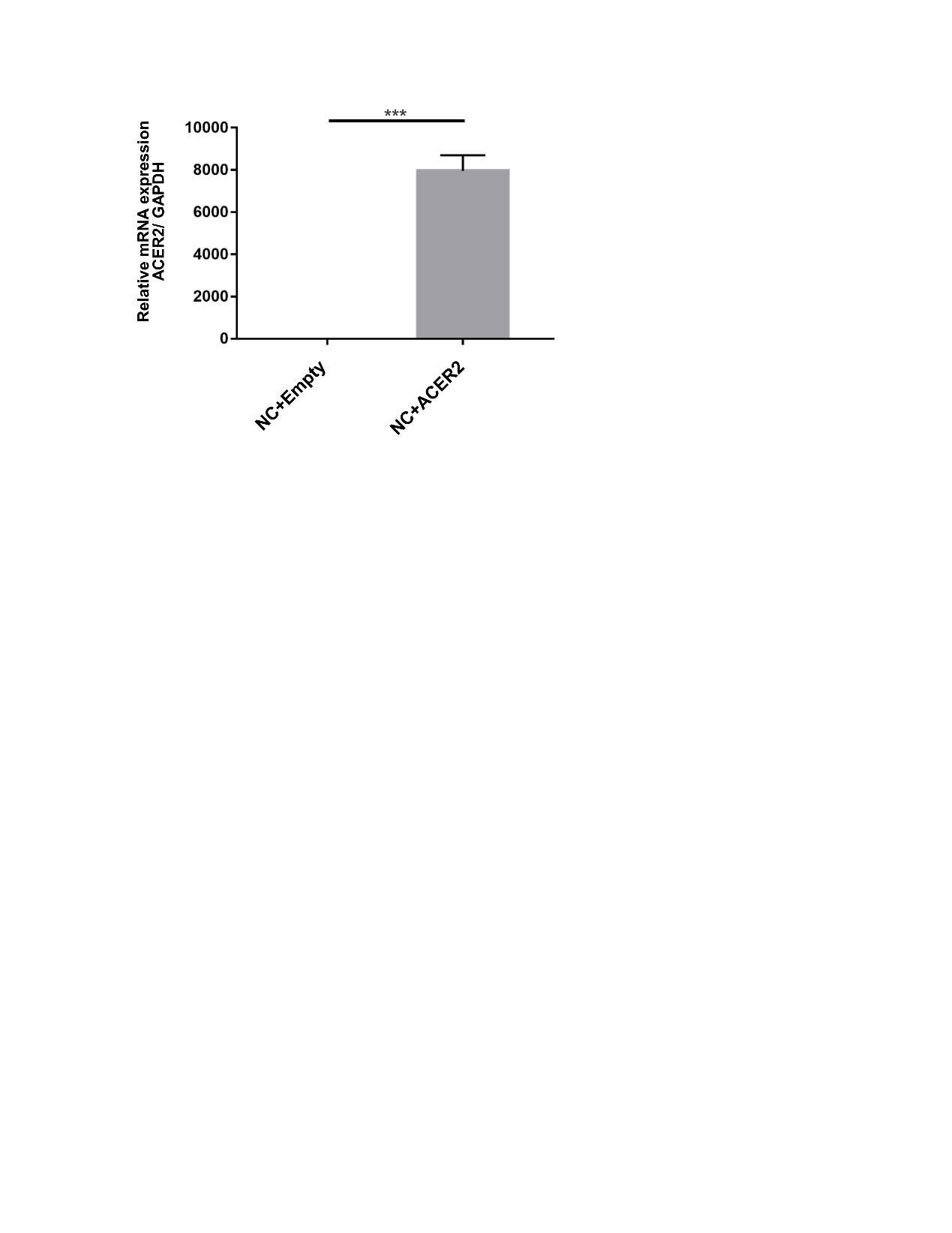


**Figure S10.** The relative mRNA expression of ACER2.


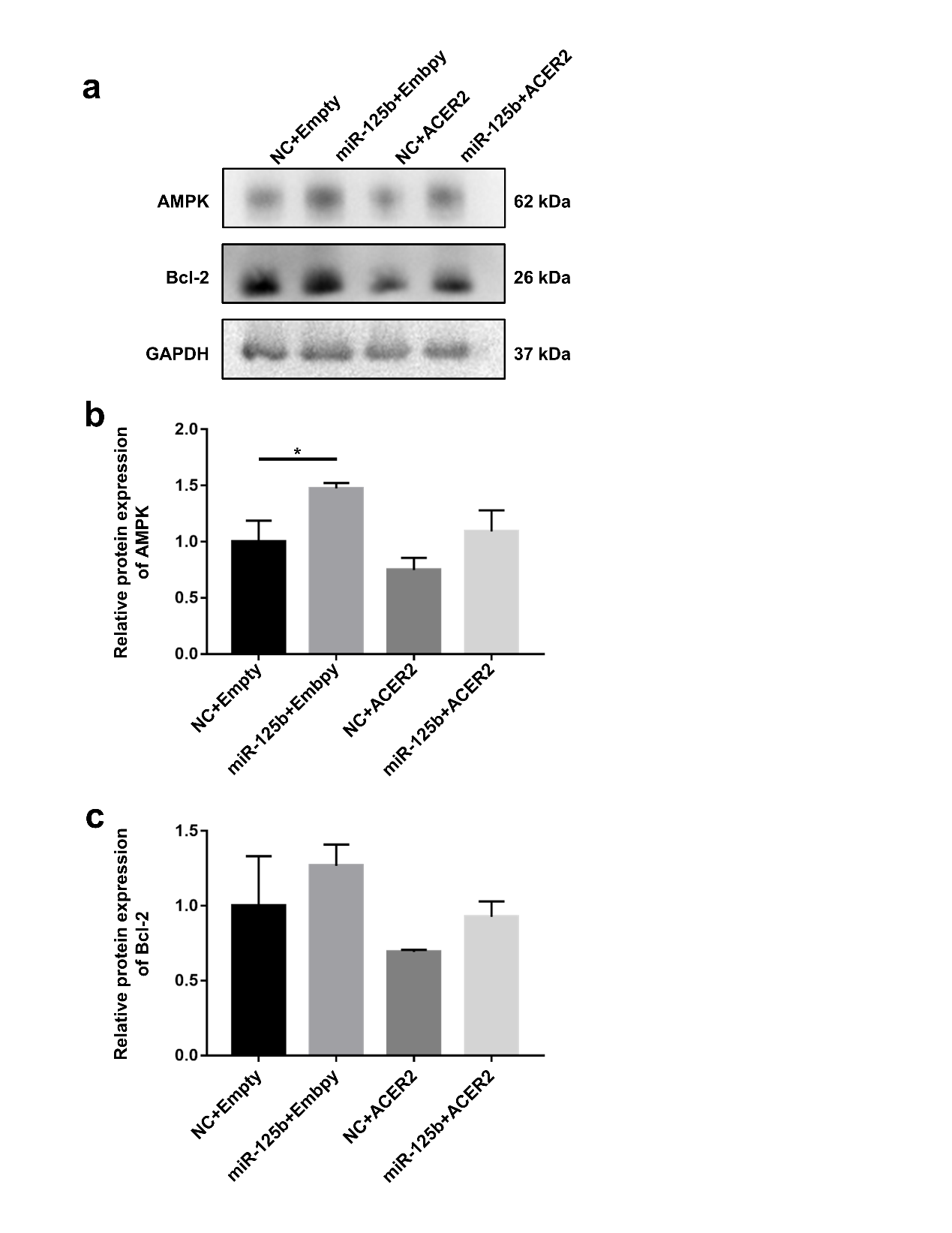


**Figure S11.** **a** Western blotting analysis of AMPK and bcl-2 protein expression in C2C12 cells in four groups. **b, c** Relative protein expression of AMPK and Bcl-2.
